# Biomineralisation by earthworms – an investigation into the stability and distribution of amorphous calcium carbonate

**DOI:** 10.1186/s12932-015-0019-z

**Published:** 2015-04-28

**Authors:** Mark E Hodson, Liane G Benning, Bea Demarchi, Kirsty E H Penkman, Juan D Rodriguez-Blanco, Paul F Schofield, Emma A A Versteegh

**Affiliations:** Environment Department, University of York, YO10 5DD York, UK; Cohen Laboratories, School of Earth and Environment, University of Leeds, LS2 9JT Leeds, UK; GFZ German Research Centre for Geosciences, Helmholtz Centre Potsdam, Telegrafenberg, 14473 Potsdam, Germany; BioArCh, Departments of Chemistry and Archaeology, University of York, York, UK; Nano-Science Center, Department of Chemistry, University of Copenhagen, 2100 Copenhagen, Denmark; Mineral and Planetary Sciences, Department of Earth Sciences, Natural History Museum, London, SW7 5BD UK; Soil Research Centre, Department of Geography and Environmental Science, School of Archaeology, Geography and Environmental Science, University of Reading, Wokingham, RG6 6DW UK; NASA Jet Propulsion Laboratory, California Institute of Technology, 4800 Oak Grove Drive, Pasadena, CA 91109 USA

**Keywords:** Calcite, ACC, CaCO_3_, FTIR, Synchrotron, Amino acids, Earthworms, Stability

## Abstract

**Background:**

Many biominerals form from amorphous calcium carbonate (ACC), but this phase is highly unstable when synthesised in its pure form inorganically. Several species of earthworm secrete calcium carbonate granules which contain highly stable ACC. We analysed the milky fluid from which granules form and solid granules for amino acid (by liquid chromatography) and functional group (by Fourier transform infrared (FTIR) spectroscopy) compositions. Granule elemental composition was determined using inductively coupled plasma-optical emission spectroscopy (ICP-OES) and electron microprobe analysis (EMPA). Mass of ACC present in solid granules was quantified using FTIR and compared to granule elemental and amino acid compositions. Bulk analysis of granules was of powdered bulk material. Spatially resolved analysis was of thin sections of granules using synchrotron-based μ-FTIR and EMPA electron microprobe analysis.

**Results:**

The milky fluid from which granules form is amino acid-rich (≤ 136 ± 3 nmol mg^−1^ (n = 3; ± std dev) per individual amino acid); the CaCO_3_ phase present is ACC. Even four years after production, granules contain ACC. No correlation exists between mass of ACC present and granule elemental composition. Granule amino acid concentrations correlate well with ACC content (r ≥ 0.7, p ≤ 0.05) consistent with a role for amino acids (or the proteins they make up) in ACC stabilisation. Intra-granule variation in ACC (RSD = 16%) and amino acid concentration (RSD = 22–35%) was high for granules produced by the same earthworm. Maps of ACC distribution produced using synchrotron-based μ-FTIR mapping of granule thin sections and the relative intensity of the ν_2_: ν_4_ peak ratio, cluster analysis and component regression using ACC and calcite standards showed similar spatial distributions of likely ACC-rich and calcite-rich areas. We could not identify organic peaks in the μ-FTIR spectra and thus could not determine whether ACC-rich domains also had relatively high amino acid concentrations. No correlation exists between ACC distribution and elemental concentrations determined by EMPA.

**Conclusions:**

ACC present in earthworm CaCO_3_ granules is highly stable. Our results suggest a role for amino acids (or proteins) in this stability. We see no evidence for stabilisation of ACC by incorporation of inorganic components.

**Graphical abstract:**
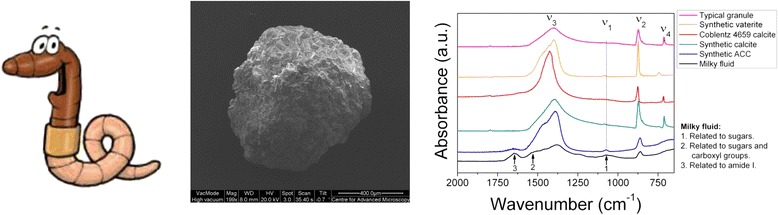
Synchrotron-based μ-FTIR mapping was used to determine the spatial distribution of amorphous calcium carbonate in earthworm-produced CaCO_3_ granules.

**Electronic supplementary material:**

The online version of this article (doi:10.1186/s12932-015-0019-z) contains supplementary material, which is available to authorized users.

## Background

Approximately 20% of all biominerals are amorphous [1] and many of the crystalline ones form through amorphous precursors [2-4]. The most common amorphous phases are silica and amorphous calcium phosphate, but amorphous calcium carbonate (ACC) is increasingly being detected on the basis of its infrared or Raman characteristics, the lack of electron diffraction patterns in transmission electron microscopy (TEM) and / or through identification of its characteristic Ca bonding environments using X-ray absorption spectroscopy (XAS) measurements (e.g. [5-8]). Pure abiogenic ACC is highly unstable, transforming within minutes to the crystalline calcium carbonate polymorphs calcite, aragonite or vaterite [9-14]. Vaterite and aragonite are metastable under most surface conditions and transform to calcite (e.g. [11,12,15]). Despite its short life span in inorganic systems ACC has been reported from organisms as diverse as higher order plants, (where it is present as cystoliths in the leaves), [16], crustaceans [17], and ascidians or sea squirts [18]. Results from the characterisation of various forms of biological ACC, and from inorganic experiments, suggest that the ACC present in biominerals may be stabilised by a variety of constituents and mechanisms. The magnesium ion has a higher hydration energy than the calcium ion; incorporation of Mg into ACC during its formation inhibits the dehydration stage during its crystallisation [19-21]. Phosphate is also known to become incorporated into ACC, either as a coating around ACC domains or within the ACC framework [22] and also inhibits its transformation to calcite [23-26]. Finally, it is also well known that a wide range of organic macromolecules including polysaccharides and proteins rich in the amino acids glutamic acid, aspartic acid, serine and glycine bind to the surface of ACC, isolating it from fluids and inhibiting its dissolution and crystallisation; such molecules can also bind strongly to free Ca^2+^ inhibiting or retarding its interaction with HCO_3_^−^ and CO_3_^2−^ during ACC formation and transformation [19,27-34].

Many species of earthworm secrete granules of calcium carbonate [35,36]. In granule-producing species the granules are formed within the earthworm’s calciferous glands. These glands contain a milky fluid that is a suspension of micron-scale ACC spherulites. As these spherulites pass through the glands they coalesce and crystallise forming millimetre-scale granules [37,38], dominantly composed of calcite but also containing remnant amorphous calcium carbonate and also vaterite and aragonite [38-42]. The granules, although much larger in size, have nevertheless the same spherical morphology as aggregates of calcite crystals produced in laboratory experiments that form when ACC precipitates from highly oversaturated solutions and crystallises to calcite via a spherulitic growth mechanism (e.g. [15,43]). Once formed from the ACC-rich milky fluid, the earthworm calcite granules are transferred from the glands into the earthworm oesophagus and from there they move down the intestine and are ultimately expelled into the soil.

Typically the ACC present in biominerals appears to fulfil one of two functions – either a rapidly accessible store of Ca for skeletal growth, or the production of mechanically robust, sometimes complex, skeletal architectures [1]. However, neither of these functions seem likely for earthworm calcium carbonate granules; rather the calcium carbonate production appears to be related to pH regulation [44-48] with calcium carbonate precipitating when HCO_3_^−^ ions are in excess of those required to buffer tissue fluid pH. Thus the granules could be viewed as an excretory product.

Analyses of granules recovered from soil up to 28 days post-expulsion and stored in dry conditions for several months to years indicate that they can still contain significant quantities of ACC, suggesting that this ACC is unusually stable compared to synthetic ACC [49]. The mechanism by which the ACC in earthworm granules is stabilised is not currently known. The purpose of the present investigation was therefore to determine whether the inorganic trace element chemistry of granules or the concentration of organic molecules present within earthworm-derived carbonate granules could account for the stability of the ACC. Our approach was to characterise the ACC content of granules and their chemical characteristics and to look for correlations between the two. We combined bulk analysis with spatially explicit analyses to address both compositional variation between granules formed by earthworms cultivated in different soils and inter- and intra-granular variation present in granules produced by individual earthworms. Additionally we carried out liquid chromatography and Fourier transform infrared (FTIR) spectroscopy to investigate the composition of the milky fluid from which the granules form. While FTIR can indicate the presence of organic material such as sugars and proteins, we chose to undertake more detailed analysis on the amino acids using liquid chromatography given the high levels of amino acids reported in studies carried out on other ACC-rich biominerals e.g. [18,50,51].

## Materials and methods

### Whole granule analysis

Earthworm-secreted calcium carbonate granules obtained in 2008 [52,53] were used in this study. In brief, 11 soils of differing properties were collected from around Berkshire, UK (see Additional file 1: Table S1). The soils were air-dried, sieved to < 250 μm and remoistened to a water content equal to 65% of their water holding capacity. Individual clitellate *Lumbricus terrestris* earthworms were kept in 300 g (dry weight equivalent) of soil for 27 days then the soils were wet sieved at 500 μm to remove freshly produced granules. There were six replicate treatments per soil and the granules produced in each of the six replicates were pooled for analysis. No granules were produced by earthworms kept in the most acidic soil (St Albans Wood, pH 4.3), leaving 10 sets of granules for characterisation. These granules are referred to in this study as “bulked granules”. Unless otherwise stated all the analyses reported in this paper relating to these granules are part of the current study and have not been reported elsewhere. For the current study the bulked granules (0.0410–0.1401 g of material) were digested in 10 mL of 5% analytical grade HNO_3_ and analysed by inductively coupled plasma-optical emission spectroscopy (ICP-OES) for a whole suite of elements.

**Figure 1:**
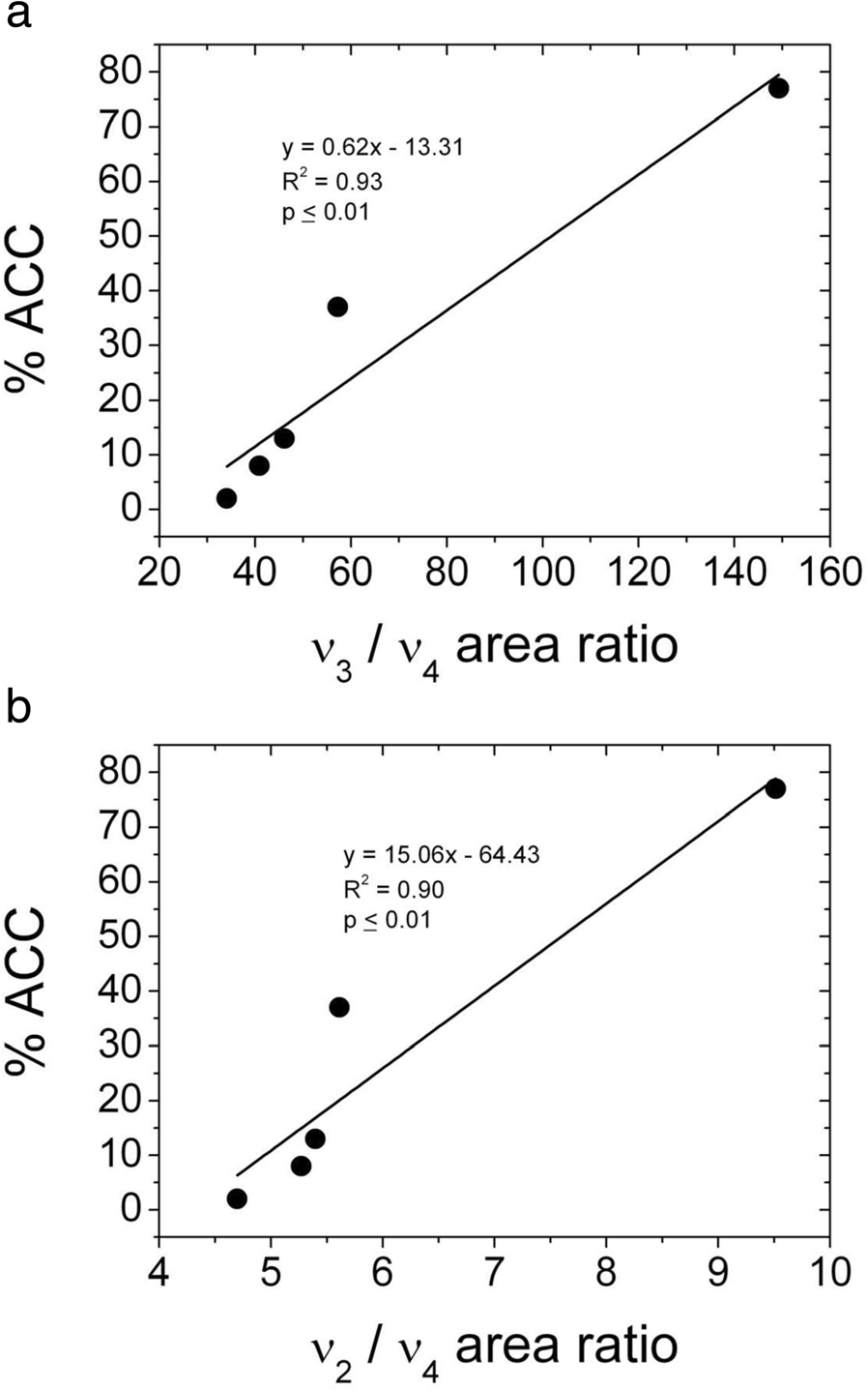
FTIR calibration curve for ACC-calcite mixtures. **a)** ratio of ν_3_ (1603–1243 cm^−1^) to ν_4_ (725–651 cm^−1^) FTIR spectra peak areas, **b)** ratio of ν_2_ (888–849 cm^−1^) to ν_4_ (725–651 cm^−1^). FTIR spectra peak areas measured on synthetic mixtures of pure ACC and calcite.

Two years after collection, i.e. in 2010, several granules from each of the bulked granule samples were gently powdered and analysed by Fourier transform infrared spectroscopy (FTIR). Calcite, and other crystalline polymorphs of calcium carbonate, have distinct bands at 714 cm^−1^ (ν_4_), 866 cm^−1^ (ν_2_), a small band at 1084 cm^−1^ (ν_1_) and a large vibration between 1420–1470 cm^−1^ (ν_3_) whilst ACC is characterized by a lack of the vibration at 714 cm^−1^ [18,38,54]. Spectra were obtained in the range 650–4000 cm^−1^ using an A2-Technology MicroLab Portable mid-IR spectrometer with a diamond internal reflection (DATR). Each spectrum comprised 512 scans with a 4 cm^−1^ resolution. The Thermo Nicolet OMNIC ESP5.1 software package was used to manipulate the spectra, including baseline subtractions, and to quantify the peak areas for all the samples. Calibration curves were then applied to determine the amount of ACC in each analysed powder. Following the 2010 analysis the powders were recovered, stored in a refrigerator at c. 6°C and re-analysed in an identical fashion in 2012 (four years after collection) except that this time each sample was analysed in triplicate to evaluate uncertainties. To produce the calibration curves, calcite and ACC were synthesised following the methods of Rodriguez-Blanco et al. [55] and Rodriguez-Blanco et al. [12] respectively. ACC:calcite mixtures in the mass proportions: 2:98, 8:92, 13:87, 37:63 and 78:22 were analysed by FTIR as described above. Peak areas for the ν_4_, ν_2_ and ν_3_ peaks covering the wavenumber ranges between ~ 650–725 cm^−1^, 850–890 cm^−1^ and 1245–1600 cm^-1^, respectively were used to produce calibration curves for % by mass ACC against the ν_3_: ν_4_ and ν_2_: ν_4_ peak area ratios (Figure 1, Additional file 2).

In 2012 a further 7 granules from the bulked granule samples (collected four years previously in 2008) from the Hamble, St Albans Field and Soil Science soils were each individually analysed by FTIR for their ACC content following the same method as above. We also dissected out the calciferous glands from earthworms cultivated in the Hamble soil for 28 days and collected the milky fluid for bulk FTIR analysis.

Following the FTIR bulk analysis of the powders for % ACC in 2010 and 2012, these same powders were used for amino acid analysis in 2013. In addition, a further 5 individual granules each from the 2008 bulk granule Hamble, St Albans Field and Soil Science soil samples that had not been previously analysed by FTIR, together with 3 milky fluid samples extracted from the calciferous glands of earthworms cultivated in the Hamble soil, were also analysed for their amino acid content.

The intra-crystalline fraction of protein in the granules was isolated by bleaching for 48 h with 12% NaOCl following the methods of Penkman et al. [56]; no bleaching was undertaken on the milky fluid samples due to the lack of biomineral. Amino acids were extracted by demineralisation of the powders and/or individual granules followed by hydrolysis of the peptide bonds in strong acid (7 M HCl, 110°C, for 24 h). During the hydrolysis step, both asparagine (Asn) and glutamine (Gln) undergo irreversible deamidation to aspartic acid (Asp) and glutamic acid (Glu), respectively [57]. The acid was evaporated to dryness and the amino acid pellet resuspended in the standard rehydration fluid used in the NEaar laboratory, which contains an internal standard (the non-protein amino acid L-homo-arginine, 0.01 mM) for quantification. Rehydrated samples were analysed by reverse-phase high-pressure liquid chromatography (RP-HPLC) following a method modified after Kaufman and Manley [58]. This method allows the quantification of the L- and D- enantiomers of aspartic acid/asparagine (Asx), glutamic acid/glutamine (Glx), serine (Ser), alanine (Ala), tyrosine (Tyr), valine (Val), methionine (Met), phenylalanine (Phe), leucine (Leu), and isoleucine (Ile). Glycine (Gly), L-histidine (L-His) and L-threonine (L-Thr) are also detected. Procedural blanks and standards of known D/L values and concentrations were randomly interspersed with samples to allow calculation of the limit of detection (LOD) and monitoring of instrument performance. The two sets of bleached samples (bulked powders and individual granules) were prepared and analysed in separate batches by RP-HPLC and the LOD was calculated for each batch, using the average concentration of amino acids found in procedural blanks: LOD = [average blanks] × 3.

Previous X-ray diffraction indicated that the granules were predominantly calcite [53]. For the current study we repeated this bulk XRD analysis in 2014 six years after collecting the granules, on powders produced by crushing several of the bulk granules. XRD scans were collected in reflection geometry with samples placed on a flat plate (zero-background holders) using a Bruker D8 Discover with CuKα radiation over a 2θ range of 3–90°(0.01°/step and 1 s/step). Patterns were compared to the standard mineral files compiled in the PDF2 database (ICDD PDF-2 Powder Diffraction File database) using the software EVA from Bruker. Phase quantification (weight %) was carried out with pattern-matching refinement of the crystalline phases using the Rietveld refinement software TOPAS [59].

### Spatially resolved analysis

For the spatially resolved analyses a new set of granules were produced in 2013. Mature, clitellate *Lumbricus terrestris* were obtained from Worms Direct (Drylands, Ulting, Nr Maldon, Essex, CM9 6QS, UK). Individual earthworms were cultured in 300 g (dry weight equivalent) < 250 μm Hamble soil to which 100 mL of deionised water had been added (see [53] and Additional file 1: Table S1 for a full characterisation of this soil). The earthworms were kept in the soil for 39 days. Subsequently the soil was wet sieved to < 500 μm to recover granules and the granules were air-dried. The earthworms were transferred to individual petri dishes lined with moist tissue paper and left for 24 hours to depurate. The soil egested by the earthworms in this period was wet sieved at 500 μm and any granules were collected and air-dried. In total we used nine earthworms to produce granules. Two “old” granules (i.e. the granules had resided in the soil for up to 39 days) were collected from the bulk soil by sieving and two “fresh” granules (i.e. the granules had been secreted within the last 24 hours) were collected from the depurate secreted by the same randomly selected earthworm. These granules were prepared for the spatially resolved analyses. Hereafter, the “old” granules are referred to as Old-1 and Old-2, and the “fresh” granules are referred to as Fresh-1 and Fresh-2.

In order to avoid the use of resin and any potential subsequent organic contamination we attempted to prepare sections of granules for spatial analysis using either a microtome or a cryomicrotome. However, during trials we found the granules were too friable to be cut in this fashion, even when the granules were covered in adhesive or held in epoxy resin. Therefore polished sections were prepared by embedding the granules in EpoFIX (Struers) resin blocks, which were ground on a coarse diamond wheel to expose the centre of the granule. The exposed surfaces were mechanically polished for 3–5 minutes using a 0.3 μm particle size corundum slurry. The bases of the resin blocks were subsequently ground until the total thickness of the block was ~3.15 mm.

The spatial distribution of ACC in the Old-1, Old-2, Fresh-1 and Fresh-2 granules was evaluated through synchrotron-based μ-FTIR maps acquired at Beamline B22 (Multimode InfraRed Imaging And Microspectroscopy, MIRIAM) of the Diamond Light Source on a Bruker Vertex 80 V FTIR instrument connected to a Hyperion 3000 microscope. Spectra were collected in reflectance mode with a liquid N_2_ cooled mercury-cadmium-telluride broadband detector at a resolution of 4 cm^−1^ by co-adding between 128 and 1024 scans per point. Large-scale maps (c. 650 μm × 760 μm, aperture 25 × 25 μm) were produced for the Old-1 and Old-2 granules using an internal globar source and a gold mirror as a reference. For the Fresh-1 and Fresh-2 granules, large maps (740 μm × 750 μm, aperture 20 × 20 μm) were acquired using the synchrotron source and a zinc selenide slide as a reference. After evaluating areas with potentially high ACC contents on both old and fresh granules, detailed maps (c. 100 μm × 150 μm) were produced using the synchrotron source but with a 6 × 6 μm aperture and a gold mirror reference. In all cases, step size was equal to the aperture dimensions and no oversampling was carried out.

**Figure 2:**
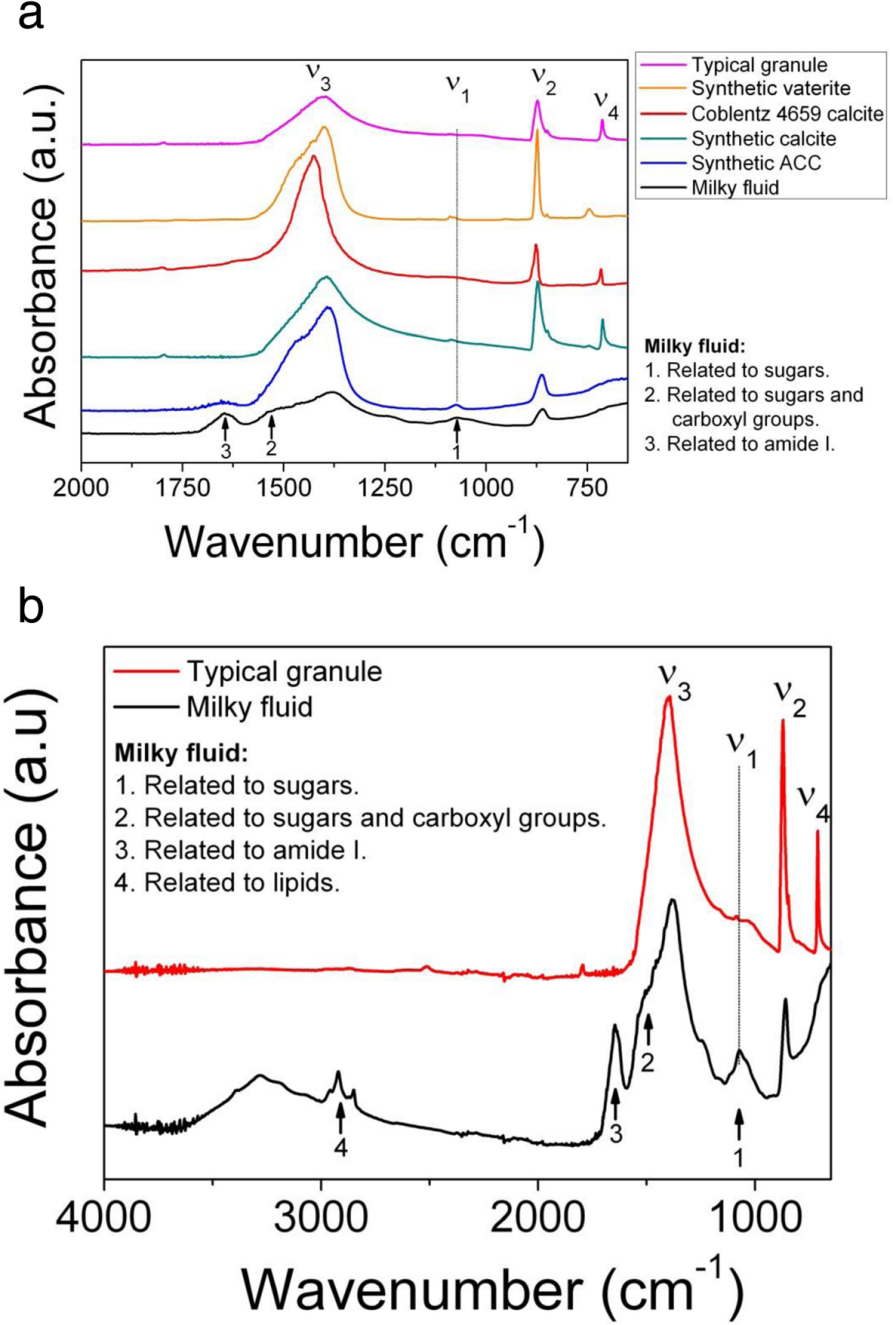
FTIR spectra of milky fluid, granules, calcite and vaterite. **a)** Finger print region (2000–650 cm^−1^) of the FTIR spectra for a typical crushed granule and the milky fluid, together with spectra for reference synthetic ACC [46], synthetic calcite , calcite (Coblentz society spectrum No. 4659) and synthetic vaterite spectra. The milky fluid and Coblentz society spectrum No. 4659 were used in the component regression carried out on the μ-FTIR spectra. Samples are offset on the vertical axis for clarity. **b)** FTIR spectra between 4000 and 650 cm^−1^ for the milky fluid and a typical granule showing the lipid peaks and the water peaks at high wavenumbers (see text). Samples are offset on the vertical axis for clarity.

The μ-FTIR data were processed using the OPUS 7.2 (Bruker) software. In the first instance the software was used to produce intensity maps for the ν_2_ and ν_4_ bands by integrating the peak areas in the ranges 855–890 cm^−1^ and 695–716 cm^−1^ respectively. Based upon previous work we utilised the fact that the presence of the ν_2_ band indicated that the analysis point was calcium carbonate, whilst the presence of the ν_4_ band indicated that the carbonate was crystalline (and, on the basis of XRD below, calcite). Absence of the ν_4_ band was taken to indicate the presence of ACC [18,38,54]. Peak area ratio maps of ν_2_ / ν_4_ were produced with high value areas indicative of ACC being the dominant carbonate phase at the analysis point (it was not possible to use the ν_3_/ν_4_ peak area ratio due to the presence of multiple peaks in the 1350–1570 cm^−1^ region, see below). We then carried out cluster analysis on the second derivative of the 855–890 cm^−1^ and 695–716 cm^−1^ regions of the spectra following vector normalization. Finally, we used standard ACC and calcite spectra (Figure 2a) to perform component regression on our spatially resolved μ-FTIR data focussing on the 650–1200 cm^−1^ wavenumber range. The standard ACC spectrum was the one obtained by analysis of the milky fluid extracted from the calciferous glands of an earthworm acquired with the above described portable A2Technology MicroLab instrument. The standard calcite spectrum was the Coblentz society spectrum No. 4659 accessed via the National Institute of Standards and Technology website [60].

The bulk mineralogy of the granules was assessed by non-destructive X-ray microdiffraction (μXRD) on the polished granules after the μ-FTIR. μXRD data were collected in reflection geometry using a Nonius PDS 120 powder diffraction system [41,61]. A 100 μm diameter beam of Cu Kα radiation was selected by a pinhole from a 300 μm diameter primary beam producing a footprint on the granule surface of ~750 × 100 μm. The probing depth is estimated to be no greater than 35 μm for these calcium carbonate granules [39]. NIST silicon powder SRM640 and silver behenate were used as external standards; calibration and data collection were performed using Diffgrab™. During data collection of at least 8000 s, the polished samples were spun continuously in the plane of the sample surface.

After the X-ray microdiffraction, elemental distribution within the granules was mapped using a Cameca SX100 electron microprobe operating at 20 kV and 100 nA with the beam set to a spot size of 1 μm. Wavelength dispersive spectrometers (WDS) were used to detect elements Mg, Mn, Fe, Na, S, P and Sr while the elements Ca, Al and Si were detected using an energy dispersive spectrometer (EDS). Maps were 512 × 512 pixels with a step size of 2 μm and dwell times of 50 μs.

## Results and discussion

### Milky fluid

The bulk FTIR spectra for the milky fluid collected from the granule-producing glands resembled that of synthetic ACC, lacking a peak at 714 cm^−1^, the characteristic peak of crystalline CaCO_3_ (Figure 2). Thus our data support the findings of Gago-Duport et al. [38], that the milky fluid is a suspension of ACC from which the predominantly calcite granules form. Similar to the data reported in [38] the spectra we collected from the milky fluid show a peak at c. 1650 cm^−1^ typical of the amide I group [62]. Although CaCO_3_ spectra have a peak at c. 1100 cm^−1^ (ν_1_), this is generally a relatively small peak whereas the milky fluid spectrum (but not the granule) has a significant peak here that could be due to sugars [62]. There is also a shoulder on the high wavenumber side of the ν_3_ peak in the milky fluid spectrum that may correspond to a peak at c. 1560 cm^−1^ that Gago-Duport et al. [38] attribute to sugars and carboxyl groups. Finally there is a distinct peak at c. 2900 cm^-1^ in the milky fluid spectrum (Figure 2b) indicating the presence of lipids [62]. Further analysis of amino acids in the granules by RP-HPLC confirmed the presence of high concentrations of amino acids in the milky fluid (Table 1). Asx, Glx, Gly, Ala and Leu were the most abundant amino acids in all three samples analysed.

**Table 1:** **Amino acid composition of milky fluid extracted from the calciferous gland of** ***Lumbricus terrestris*** **earthworms**

**Sample number**	**1**	**2**	**3**
Amino acid content/picomol mg^−1^			
Asx	53914 ± 33	82116 ± 489	126484 ± 3622
Glx	59950 ± 218	89784 ± 1218	135972 ± 2598
Ser	33449 ± 151	57377 ± 1005	77156 ± 1266
L Thr	26546 ± 35	42055 ± 428	58750 ± 1860
L His	16640 ± 668	26532 ± 1338	44539 ± 106
Gly	55022 ± 1808	106161 ± 4433	128852 ± 1961
L Arg	25831 ± 159	58534 ± 528	59731 ± 1632
Ala	43439 ± 25	70961 ± 422	101471 ± 3208
Tyr	12024 ± 209	20348 ± 354	21983 ± 604
Val	31258 ± 168	49848 ± 510	73772 ± 2197
Phe	20818 ± 68	32369 ± 296	50379 ± 1857
Leu	50905 ± 386	74546 ± 466	119497 ± 3190
Ile	26320 ± 179	39651 ± 119	63205 ± 1371
Met	358 ± 80	610 ± 98	1242 ± 614

The error term is one standard deviation about the mean of duplicate analyses. LODs are on the order of 1–60 picomoles mg^−1^ for each amino acid.

### Granules

X-ray diffraction of the powdered bulked granule samples for this study confirmed our previous results [53] that the granules are predominantly calcite; trace quartz and vaterite were also detected (Additional file 3: Table S2, Additional file 4: Figure S1). The quartz is most likely due to inclusions in the granules [49]. We were unable to detect the diffuse peaks indicative of ACC on the XRD traces [12] as the crystallinity of the calcite and quartz dominated the intensities in the traces. The presence of vaterite over 6 years since granule production confirms that “unstable” polymorphs of CaCO_3_ are stable in the granules. As vaterite was only present at trace levels and the bulk FTIR spectra of calcite and vaterite are almost identical (Figure 2), we did not try and distinguish between these two phases when quantifying granule composition from the FTIR spectra obtained from the analysis of the bulk granules and the spatial analysis (see below). Thus, in a sense our FTIR “calcite” below is actually representative of both crystalline CaCO_3_ polymorphs, vaterite and calcite.

The elemental composition, the bulk FTIR peak area ratios and calculated % ACC, and the amino acid contents of the bulk granule samples are given in Tables 2, 3 and 4 (see Additional file 2 for typical FTIR spectra). FTIR analysis of the bulk granules was carried out in 2010 and again in 2012. Previously we have detected good correlations between the trace element concentrations of granules and the concentrations in the soils in which the earthworms producing the granules were kept [39-41]. However the only significant correlation found in the current study was between soil exchangeable Sr (Additional file 1: Table S1) and granule Sr (r = 0.70, p ≤ 0.05, Rank Spearman correlation), possibly due to the restricted range of soil concentrations used, or the use of real soils. Our previous studies were mainly carried out on amended soils and it is well established that the availability of metals in such soils is far higher than it is in real soils (e.g. [63,64]).

**Table 2:** **Granule elemental composition**

**Sample name**	**Coombe**	**Frilsham**	**Hamble**	**Kettering**	**Neville**	**Parkgate**	**Soil Science**	**St Albans field**	**Tidmarsh**	**Wilderness**	**LOD**
Elemental composition/mg kg^−1^											
Ag	2.56	2.30	<2.02	<2.02	2.37	3.73	3.02	2.63	<2.02	2.76	2.02
Al	135	190	133	127	96.0	244	171	172	83.7	133	44.4
Cu	2.12	2.01	2.97	<1.96	3.18	6.59	3.83	<1.96	<1.96	2.81	1.96
Fe	138	277	262	170	289	355	141	658	117	168	5.13
Li	0.76	0.28	0.57	0.81	0.54	0.81	0.47	0.63	0.37	0.39	0.11
Mg	929	473	479	863	644	602	596	710	446	420	0.50
Mn	326	386	1390	529	554	870	305	433	337	642	1.00
Na	870	632	664	668	834	1880	779	682	591	743	5.47
P	1640	149	<114	<114	249	254	<114	188	162	<114	114
Pb	<7.40	<7.40	<7.40	<7.40	<7.40	<7.40	185	<7.40	<7.40	371	7.40
Sr	482	352	419	437	491	546	550	528	383	640	0.16
Zn	11.7	13.2	5.82	4.54	57.0	70.5	14.2	81.9	9.04	36.7	0.79

Determined on an acid digest of the bulked granules obtained in 2008 (0.04–0.14 g material) followed by analysis by ICP-OES.

Additional analytes that were below detection (LOD in brackets) were B (11.8 mg kg^−1^), Bi (71.7 mg kg^−1^), Cd (0.86 mg kg^−1^), Co (1.40 mg kg^−1^), Cr (2.61 mg kg^−1^), Ga (12.5 mg kg^−1^), In (27.3 mg kg^−1^), K (248 mg kg^−1^), Ni (1.37 mg kg^−1^), Tl (145 mg kg^−1^).

**Table 3:** **Granule ACC content (same samples as used for amino acid analysis, Table** **4** **)**

**Sample name**	**Coombe**	**Frilsham**	**Hamble**	**Kettering**	**Neville**	**Parkgate**	**Soil science**	**St Albans field**	**Tidmarsh**	**Willderness**
2010										
ν_3_: ν_4_ areas	27.92	28.09	27.92	30.27	32.00	30.77	32.61	25.12	29.79	29.38
% ACC (ν_3_: ν_4_)	4.0	4.1	4.0	5.5	6.6	5.8	7.0	2.3	5.2	5
ν_2_: ν_4_ areas	4.07	4.11	4.10	4.42	4.00	4.50	4.91	3.45	4.48	4.06
% ACC (ν_2_: ν_4_)	−3.2	−2.5	−2.8	2.1	−4.3	3.3	9.5	−12.4	3.1	−3.4
2012										
ν_3_: ν_4_ areas	24.26 ± 0.39	25.82 ± 0.95	23.78 ± 1.14	25.09 ± 2.23	21.07 ± 0.49	27.68 ± 1.73	26.00 ± 1.15	20.33 ± 0.54	23.62 ± 1.40	23.41 ± 2.55
% ACC (ν_3_: ν_4_)	1.73 ± 0.24	2.70 ± 0.59	1.43 ± 0.71	2.25 ± 1.39	−0.25 ± 0.30	3.85 ± 1.07	2.81 ± 0.72	−0.71 ± 0.33	1.33 ± 0.87	1.20 ± 1.58
ν_2_: ν_4_ areas	2.68 ± 0.02	2.88 ± 0.06	2.63 ± 0.16	2.32 ± 0.06	2.32 ± 0.06	2.94 ± 0.13	2.85 ± 0.11	2.28 ± 0.02	2.59 ± 0.09	2.59 ± 0.20
% ACC (ν_2_: ν_4_)	−24.01 ± 0.30	−21.07 ± 0.88	−23.50 ± 2.40	−23.50 ± 2.40	−29.42 ± 0.95	−20.09 ± 1.90	−21.54 ± 1.67	−30.14 ± 0.32	−25.43 ± 1.39	−25.44 ± 3.08

Determined on a subsample of granules, bulked together, taken from the bulked granules obtained in 2008. ACC content was calculated using the calibration curves shown in Figure 1. With respect to the 2012 data, 3 repeat readings were made on the same sample, values are mean ± standard deviation, n = 3.

**Table 4:** **Granule amino acid composition measured in 2012 by RP-HPLC**

**Sample name**	**Coombe**	**Frilsham**	**Hamble**	**Kettering**	**Neville**	**Parkgate**	**Soil science**	**St Albans field**	**Tidmarsh**	**Wilderness**	**LOD**
**Amino acid content/pico mol mg** ^**−1**^											
Asx	144	152	207	217	102	179	210	32	177	125	10
Glx	90	107	178	138	54	151	151	24	123	54	16
Ser	79	114	177	176	103	130	152	40	117	101	25
L Thr	52	81	82	123	79	63	108	26	74	50	4
L His	8	12.	57	19	9	23	18	0	19	8	0
Gly	109	192	386	376	173	265	258	82	182	157	60
L Arg	32	45	57	55	41	47	54	14	45	25	5
Ala	65	106	161	194	95	115	152	35	107	87	10
Val	59	93	87	141	72	60	108	29	77	37	8
Phe	16	25	38	33	17	33	29	0	23	10	8
Leu	35	57	58	76	41	43	70	21	48	29	16
Ile	23	44	35	59	28	26	50	5	35	15	7
Met	5	45	57	55	41	47	54	14	45	25	5

Determined on the same powders derived from several granules per soil on which ACC content was determined, Table 3).

Granules were also analysed for Tyr but this amino acid was below the detection limit for all samples (8.00 pico mol mg^−1^).

Ignoring samples with concentrations lower than the limit of detection, the data show that whilst concentrations of most analysed elements typically varied by a factor of 5 or less P concentrations varied by a factor of 11. Phosphate has been shown to stabilise ACC [23-26]. The high level of variation of P in the granules could potentially cause significant variation in granule ACC content as well.

The FTIR spectra obtained for the powdered bulked granules resemble that of calcite (Figure 2), in support of our previous X-ray diffraction data [53] and the XRD data obtained on bulked powdered granules in this study. Despite amino acids being detected in the milky fluid and the granules by RP-HPLC (Table 4) and peaks corresponding to organic molecules being present in the milky fluid FTIR spectra, no peaks suggestive of organic molecules were seen in the spectra of obtained for the granules. This presumably reflects the sensitivity of the two techniques for detecting organic molecules. As the mechanism by which organic molecules are thought to stabilise ACC involves sorption and inhibition of dissolution and crystallisation [19,27-34] it could be that any stabilising organic molecules are present at very low concentrations, potentially as molecular monolayers. Such a signal would be heavily diluted within the bulk granules analysed by FTIR and thus hard to detect.

The ratio of the ν_3_: ν_4_ and ν_2_: ν_4_ peak areas in the granule spectra varied between samples (Table 3), potentially because of varying amounts of ACC and calcite between samples. When peak areas were entered into the calibration curve equations determined on artificial ACC:calcite mixtures (Figure 1) to quantify the % ACC present in the granules, the ν_3_: ν_4_ peak area equation consistently yielded larger values than the ν_2_: ν_4_ peak area equation and the values derived from the ν_2_: ν_4_ peak area equation were often negative (Table 3). The negative values may arise due to differences in grain size and orientation between the synthetic standards used to construct the calibration curves and the powdered granules (see for example [65]). We took great care to grind all our samples to the same degree and for a significant period of time to ensure homogeneity of the powder and significant grain size reduction. Unfortunately small sample size made the use of the grinding curve approach of [65] impractical as a check for grain size effects though our use of peak area rather than peak height may have off-set any significant effects. Typically reductions in peak height are accompanied by increasing peak width, the two changes largely cancelling each other out when peak area is assessed (as in this study). Additionally or alternatively the negative values may be due to differences in crystal properties such as crystallinity between the synthetic standards and the granule carbonates, as is often the case when comparing biogenically produced carbonates with abiogenic carbonates (e.g. [66]). However, the two sets of values are well correlated (r = 0.8, p ≤ 0.01 for the 2010 data, r = 1.0, p ≤ 0.05 for the 2012 data, Pearson correlation) suggesting that they are at least a good indicator of relative amounts of ACC. Thus the bulk FTIR analysis indicates that the granules from the different samples have varying amounts of ACC and that this ACC is long lived. The first ACC measurements were made in 2010, 2 years after extraction of the granules from the soil. The second set of measurements, carried out two years later (in 2012), yielded lower ACC concentrations thus suggesting further crystallization of the ACC. Nevertheless, the 2010 ν_2_: ν_4_ peak areas (and calculated ACC %) showed strong correlations with the 2012 ν_2_: ν_4_ peak areas (p = 0.7, p ≤ 0.05, Pearson correlation) and 2012 ν_3_: ν_4_ peak areas (p = 0.8, p ≤ 0.05, Pearson correlation). Thus, despite the reduction in ACC between 2010 and 2012, earthworm ACC appears to be stable for several years. The stability of biogenic ACC is rarely assessed on these timescales but in many cases reported in the literature, biogenic ACC appears to be associated with transient features, e.g. the formation of sea urchin spicules [7] or the storage of Ca to be used during cuticle growth following moulting in crustaceans (e.g. [67,68]. Therefore, the level of ACC stability in the earthworm granules is unprecedented and warrants further investigation.

**Figure 3:**
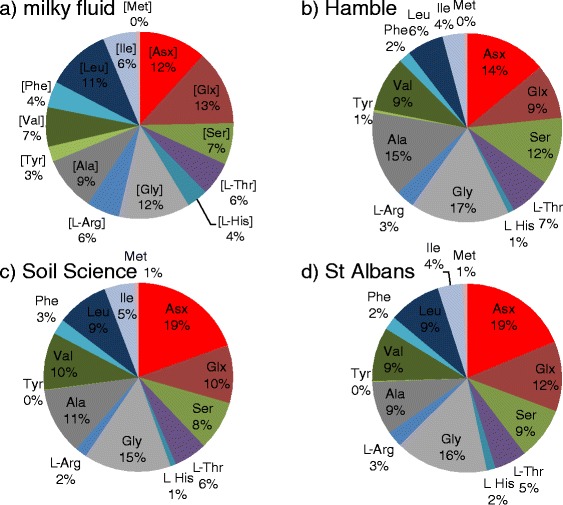
Average amino acid compositions of the **a)** milky fluid (Table 1) and **b** – **d**) granules (Table 5). Although the general compositions are similar, the percentage of Asx is significantly higher in the granules.

Following bulk FTIR analysis in 2012, the samples were analysed for their amino acid content. The intra-crystalline amino acid concentrations in the granules (Table 4) are significantly lower than those in the milky fluid (total amino acid concentrations of ~ 1 vs ~ 700 nanomol mg^−1^, Tables 1, 4, 5). but are not dissimilar to those from a range of mollusc shells [69], slightly lower than *Porites* coral [70], and significantly lower than *Bithynia* opercula [71], *Patella* shell [72] and ostrich eggshell [73]. Asx, Glx, Ser, Gly and Ala were consistently the most dominant amino acids in the granules. The relative proportions of the amino acids in the milky fluid and granules are similar although not identical. In particular, the proportion of Asx represented in the earthworm granules (14–19%) is consistently higher than that in the milky fluid (12%) (Figure 3). While the fraction of amino acids incorporated into the granules is generally representative of the overall amino acid content of the milky fluid, this result suggests that the mechanism of incorporation (or of removal by bleaching) is selective.

**Table 5:** **Mean granule amino acid (n = 5) and ACC composition (n = 7)**

**Amino acid content / pico mol mg** ^**−1**^	**Hamble**	**Soil Science**	**St Albans field**	**LOD**
Asx	217 ± 103	293 ± 74	269 ± 31	40
Glx	145 ± 80	150 ± 29	173 ± 27	59
Ser	179^a^ ± 20	128^b^ ± 33 (2)	129^b^ ± 17	107
L Thr	108^a^ ± 16	84^ab^ ± 23	78^b^ ± 11	25
L His	16 ± 1	15 ± 9 (1)	22 ± 6	5
Gly	262 ± 40 (1)	223 ± 70 (3)	228 ± 74 (3)	212
L Arg	50^a^ ± 8	29^b^ ± 14 (3)	39^ab^ ± 6 (1)	31
Ala	227^a^ ± 43	173^a^ ± 54	131^ab^ ± 17	39
Tyr	7^a^ ± 3 (2)	BD^b^	BD^ab^	6
Val	146 ± 44	148 ± 46	133 ± 24	30
Phe	31 ± 5 (1)	40 ± 12 (1)	33 ± 8 (1)	28
Leu	97 ± 33 (4)	133 ± 48 (2)	128 ± 45 (2)	103
Ile	58 ± 16	78 ± 24	61 ± 8	35
Met	BD	9 ± 5 (3)	10 ± 6 (4)	10
ν_3_: ν_4_ areas	23.15^a^ ± 0.97	27.41^b^ ± 3.24	27.91^b^ ± 1.33	
% ACC (ν_3_: ν_4_)	1.04^a^ ± 0.60	3.69^b^ ± 2.01	3.99^b^ ± 0.83	
ν_2_: ν_4_ areas	2.56^a^ ± 0.07	2.90^b^ ± 0.30	2.90^b^ ± 0.11	
% ACC (ν_2_: ν_4_)	−25.82^a^ ± 1.10	−20.69^b^ ± 4.52	−20.77^b^ ± 1.60	

Determined on sets of granules taken from the “bulk granule” sets obtained in 2008. Values are given ± standard deviation. Number in bracket after standard deviation indicates number of samples below detection (BD) limit. LOD shows limit of detection. Concentration < LOD were included in the statistical analysis.

^a,b^Across rows values with different superscripts are significantly different from each other based upon the Tukey test with p ≤ 0.05.

**Figure 4:**
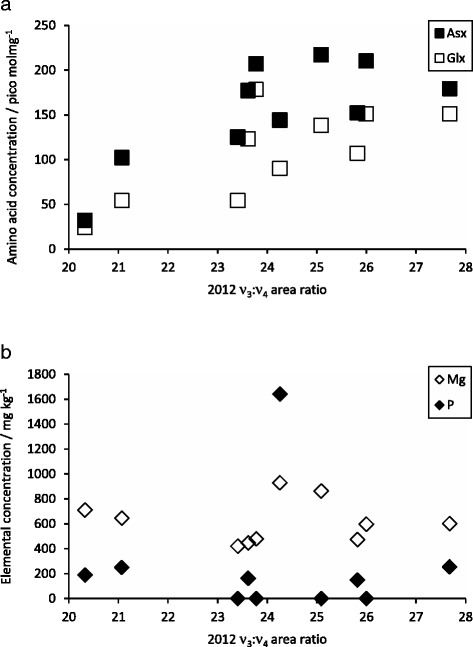
Correlations between 2012 ν_3_: ν_4_ area ratio and selected chemical components of the granules. **a)** Plot of bulk granule Asx and Glx concentrations against 2012 ν_3_: ν_4_ area ratio showing significant correlations between each amino acid and the peak area ratio (r ≥ 0.7, p ≤ 0.05). **b)** Plot of bulk granule Mg and P concentrations against 2012 ν_3_: ν_4_ area ratio showing the lack of correlation between either element and the peak area ratio.

The 2010 ν_2_: ν_4_ area ratios obtained from the FTIR spectra of the bulk granules show significant positive correlations with the majority of amino acids (r ≥ 0.7 (Pearson correlation), p ≤0.05 for Asx, Glx, Ser, L-Thr, L-Arg, Ala, Phe, Leu and Ile; r = 0.7 (Spearman rank correlation), p ≤ 0.05 for L His). The 2010 ν_3_:ν_4_ peak areas and both 2012 peak area ratios show far fewer correlations. The 2010 ν_3_: ν_4_ peak areas correlate with L-Thr (r = 0.7, p ≤ 0.05, Pearson correlation) whilst both sets of 2012 peak areas correlate with Asx, Glx and Phe (r ≥ 0.7, p ≤0.05, Pearson correlations) (e.g. Figure 4a). It is not clear why the 2010 ν_2_: ν_4_ peak areas should show so many correlations whilst the other peak area ratios do not. The change in the number of significant correlations between the 2010 and 2012 ν_2_: ν_4_ peak areas may perhaps reflect the gradual crystallisation of the ACC. It is unknown whether proteins or amino acids, or indeed other organic molecules or inorganic impurities, control the formation or stabilisation of the earthworm granules but the presence of amino acids in the milky fluid and granules and the correlations between amino acids and ACC contents are consistent with this interpretation. Similar findings are reported in the literature for ACC stabilization in biominerals produced by other ogranisms. Several studies reported aspartic acid (Asx) as stabilising ACC in synthesis experiments [27,33,74] whilst Aizenberg et al. [50] reported that the ACC in sponge and sea squirt spicules was enriched in Glx, Ser and Gly.

In 2008, when the first set of granules were collected, we determined their elemental composition by acid digestion and analysis by inductively coupled plasma-optical emission spectroscopy (ICP-OES). No significant correlations are seen between the peak area ratios or % ACC (Table 3) and granule elemental composition (Table 2) (e.g., Figure 4b). Given the wide range of P concentrations in the granules, and the role of P in stabilising ACC in some systems [22-26] we had anticipated a possible correlation between P concentration and % ACC. The lack of such a correlation might be due to the heterogeneous distribution of both phosphate and ACC within granules or be because the ACC in the granules is not stabilised by phosphate. Additionally, since the bulk FTIR and amino acid analysis were performed on the same samples, different to those used for the elemental analysis, it is perhaps not surprising that there were many significant correlations between the FTIR peak ratios and granule amino acid content and none between the ACC and the granule elemental compositions.

### Intragranule heterogeneity

In 2012 to further investigate the relationship between amino acid concentrations and ACC contents, and because of concerns regarding granule heterogeneity following our previous observations of elemental [39-41] and phase [42,49] heterogeneity in the granules, we analysed individual granules taken from the “bulked granules” sample set. Amino acid and ACC analyses were carried out on separate sets of granules due to limited sample size available for analysis. We were not able to determine the elemental variability between individual granules as the granules have too low a mass to yield solutions of appropriate volume and concentration for analysis on our ICP-OES. Granules from the Hamble, Soil Science and St Albans Field soils were selected; 7 were analysed for ACC, 5 for amino acids (Table 5).

The data are very similar to those obtained on the bulk granules (Tables 3 and 4). Individual granules have highly variable amino acid concentrations, though the relative proportions of amino acids are similar. The mean RSDs for the amino acid concentrations for the Hamble, Soil Science and St Albans granules were 26, 35 and 22% respectively. In contrast the mean RSDs for the FTIR peak area ratios (ν_2_:ν_4_ and ν_3_:ν_4_) were 6%, though the mean RSD for the calculated % ACC was higher at 16%. The FTIR peak area ratios suggest that the granules from the Soil Science and St Albans soils have higher ACC contents than those from Hamble but, where there is a significant difference, they have lower amino acid concentrations. This pattern is different from the correlations resulting from our analysis of the bulk granules (Tables 3 and 4, Figure 4a).

The lack of consistent relationships between the FTIR/ACC and amino acid data in Tables 3, 4, and 5 could be due to granule heterogeneity. An additional significant hindrance to the interpretation of the data in Table 5 is that the amino acid and ACC determinations were carried out on separate granules and that elemental analysis was not performed. For this reason we attempted spatially resolved μ-FTIR at the Diamond Light Source and combined that with elemental analysis of the same granules by electron microprobe analysis (EMPA). We hoped to be able to map out both ACC and amino acid distribution using μ-FTIR on individual granules. Bulk FTIR did not reveal the presence of organic molecules in the granules but we hoped that spatially-resolved analyses of the inherently heterogeneous granules would reveal areas of higher, detectable, concentrations of ACC and associated organic functional groups. This would then allow comparison of ACC, organic functional group and elemental distribution taking into account spatial distributions, on individual granules.

### Spatially resolved analyses

We carried out spatial analysis on 4 granules. Two granules (Old-1 and Old-2) were recovered from soil up to 39 days post secretion. The other two (Fresh-1 and Fresh-2) were recovered within 24 hours of secretion. Non-destructive μXRD on thin sections of the granules indicated that the crystalline phases present were dominantly calcite. Only calcite was detected in Fresh-1 and Fresh-2 granules. Old-1 granule contained trace amounts of aragonite and Old-2 trace amounts of quartz. Aragonite has been detected in granules previously (e.g. [38,39,42]) and the trace amounts of quartz are most likely due to inclusions present in the granules (e.g. [49]).

**Figure 5:**
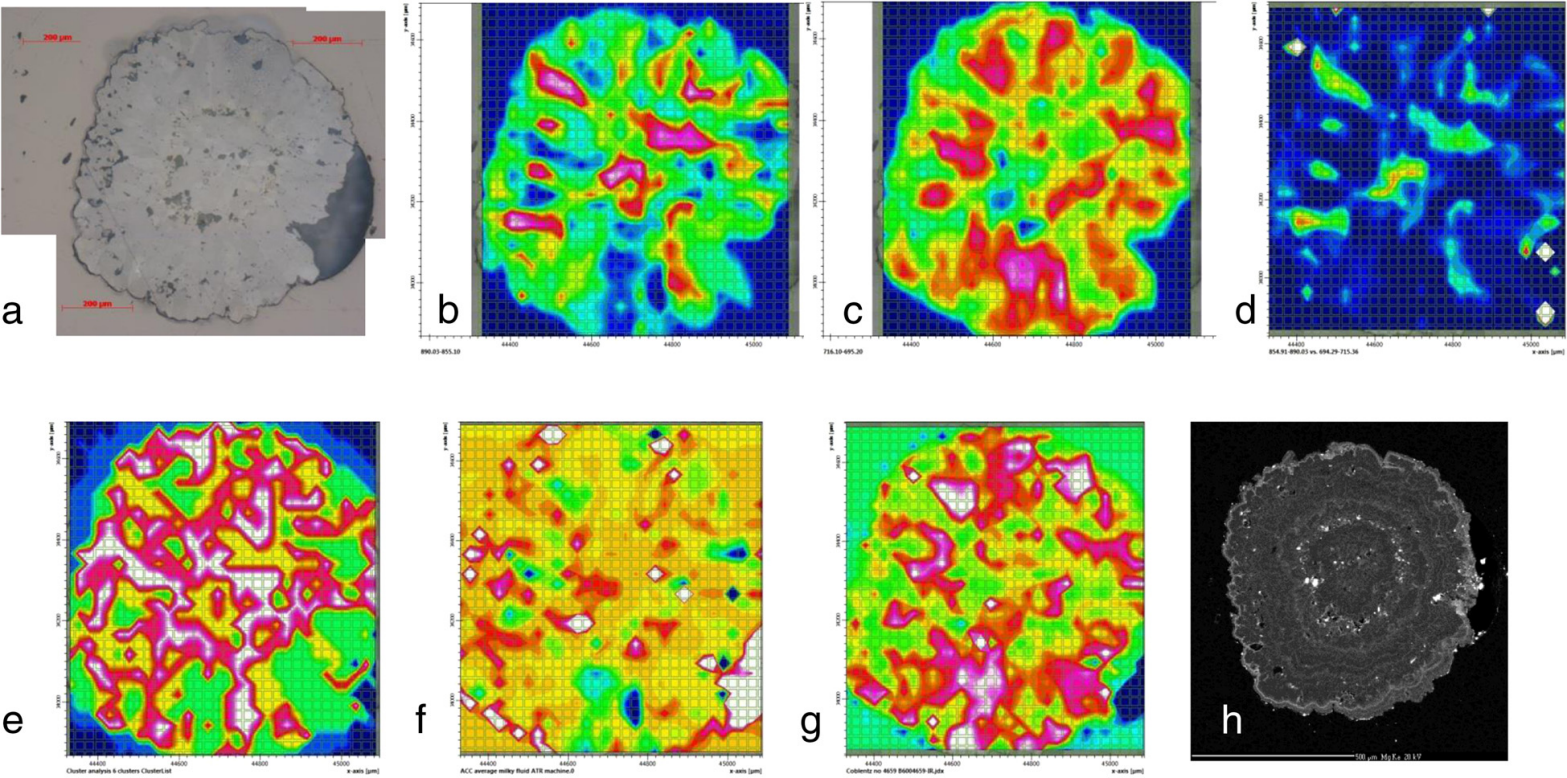
Images and intensity maps from analysis of a thin section of granule Fresh-1. **a)** reflected light image of granule Fresh-1 collected within 1 day of secretion, scale bar is 200 μm; μ-FTIR intensity maps for **(b)** ν_2_ (855–890 cm^−1^), **(c)** ν_4_ (695–716 cm^−1^) and **(d)** ν_2_/ν_4_ ratio map with high intensity areas indicative of ACC rich regions; **(e)** cluster analysis map showing 6 clusters, **f)** component regression intensity map for ACC, **g)** component regression intensity map for calcite, **h)** electron microprobe Mg distribution map showing the concentric growth features in the granule. For the μ-FTIR intensity maps, red indicates high intensity, blue indicates low intensity, while for the elemental maps bright indicates higher concentrations.

**Figure 6:**
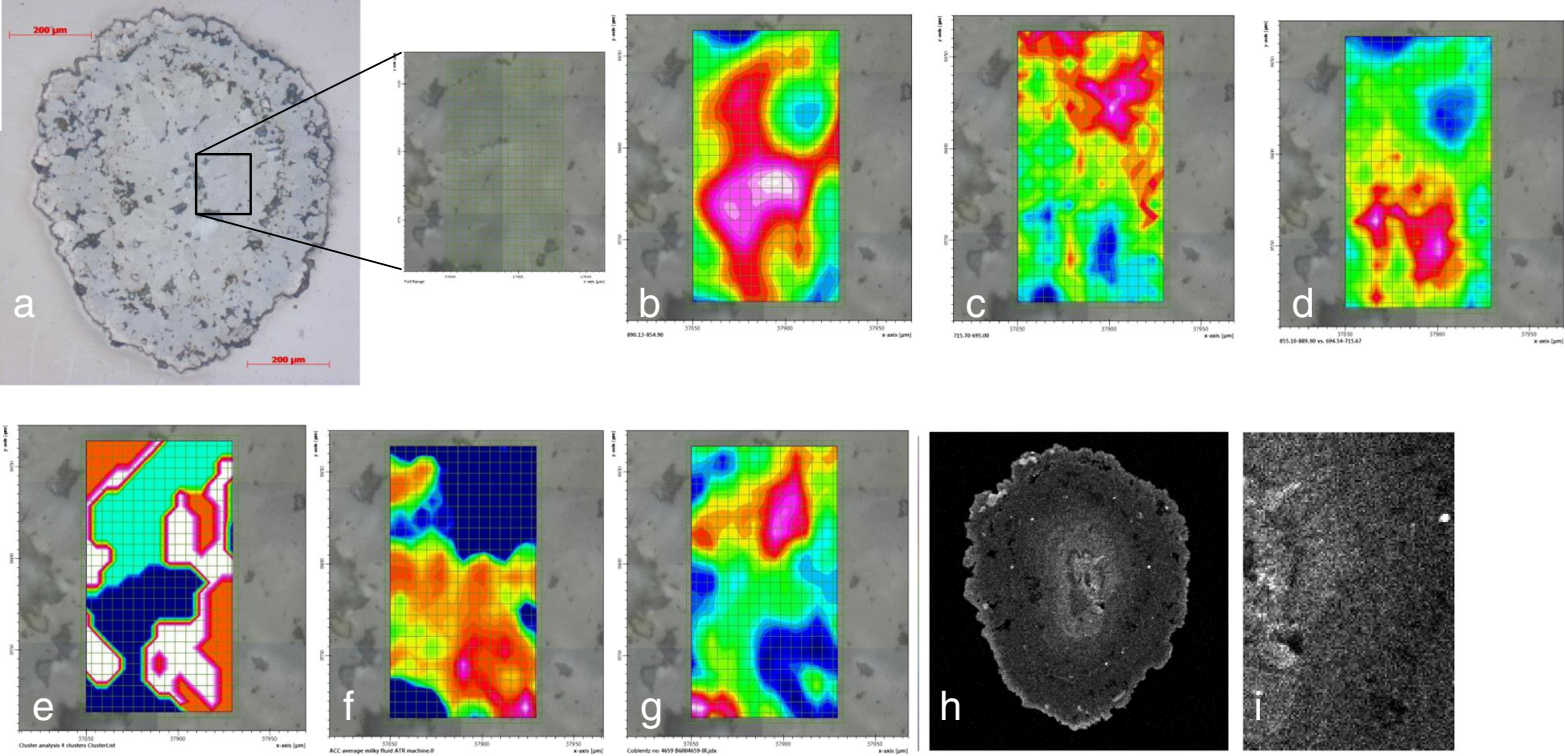
Images and intensity maps from analysis of a thin section of granule Old-2. **a)** reflected light image of an area of granule Old-2 resident in soil for between 1 and 39 days, scale bar is 100 μm; high spatial-resolution μ-FTIR intensity maps for **(b)** ν_2_ (855–890 cm^−1^), **(c)** ν_4_ (695–716 cm^−1^) and **(d)** ν_2_/ν_4_ ratio map with high intensity areas indicative of ACC rich regions, **e)** cluster analysis map showing 4 clusters (dark blue, light blue, orange and white), **f)** component regression intensity map for ACC, **g)** component regression intensity map for calcite, **h)** electron microprobe Mn distribution map, **i)** close up of **(h)** focussing on area of μ-FTIR maps showing lack of correlation between elemental and ACC variation. For μ-FTIR intensity maps, red indicates high intensity, blue indicates low intensity while for the elemental maps bright indicates higher concentrations.

**Figure 7:**
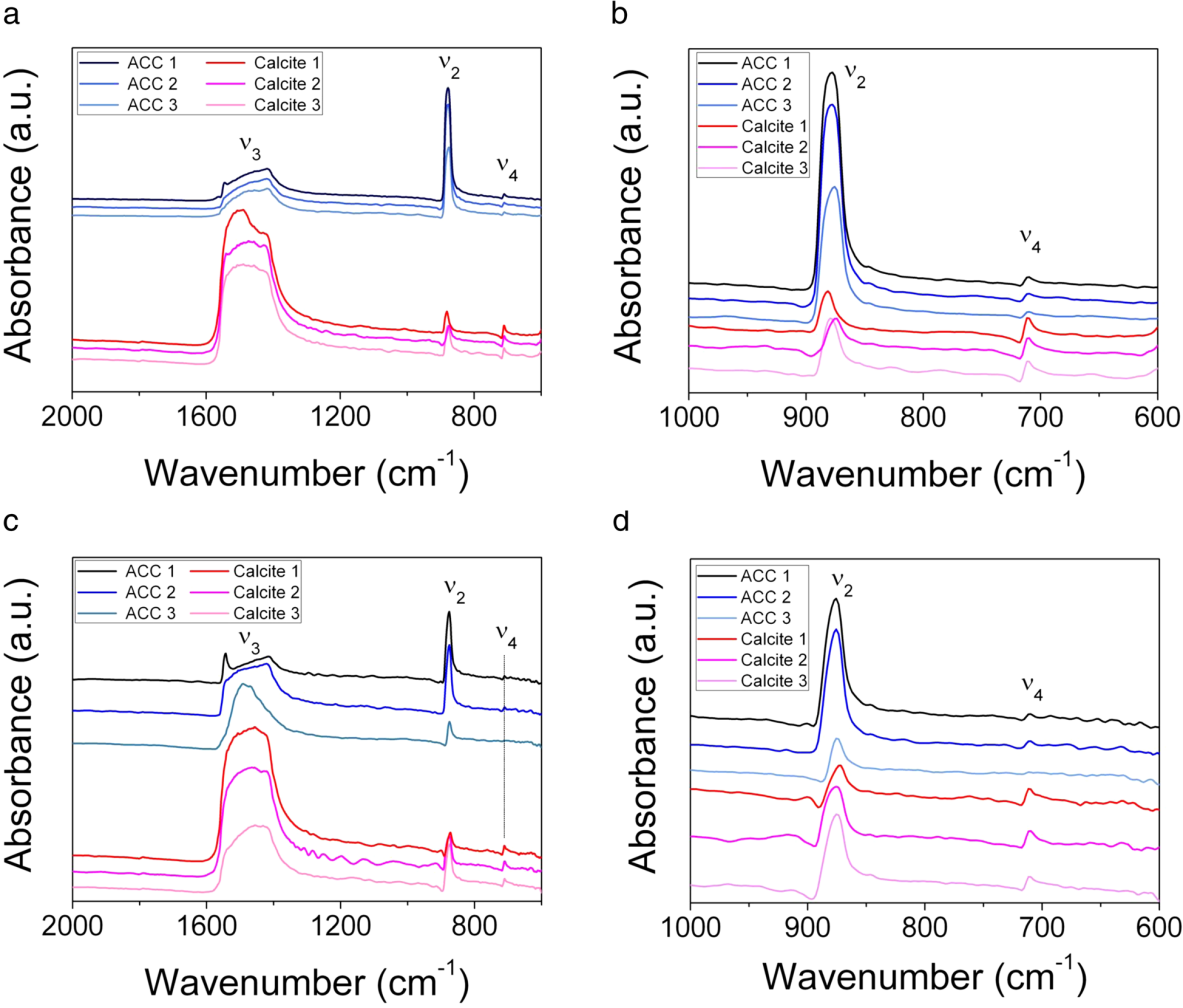
Typical μ-FTIR spectra obtained from granules. **a**, **b)** Typical μ-FTIR spectra obtained from ACC-rich and calcite-rich regions of granule Fresh-1 imaged in Figure 5; **c**, **d)** Typical μ-FTIR spectra obtained from ACC-rich and calcite-rich regions of granule Old-2 imaged in Figure 6. Samples are offset on the vertical axis for clarity.

Our initial μ-FTIR mapping of the granules indicated significant variation in the intensity of the different calcium carbonate peaks detectable, both when we mapped at a large scale (Figure 5b, c) and a small scale (Figure 6b, c) (see Additional file 5 for further intensity maps). This variation was present both in the fresh (Figure 5) and old (Figure 6) granules and suggested that significant portions of the granules could comprise ACC. FTIR spectra of calcite have a relatively low ν_2_: ν_4_ peak area ratio relative to ACC as ACC lacks a ν_4_ peak (Figure 2). Ratio maps of the ν_2_: ν_4_ peak areas indicated areas of granules likely to be ACC-rich (Figures 5d, 6d). The cluster analysis groups similar spectra together and shows the spatial distribution of these groupings. Maps of the spatial distribution of 4 to 7 clusters (Figures 5e, 6e) were required before a distribution of clusters similar to that of our manually produced wavenumber intensity peaks was achieved. Perhaps more convincing, in terms of identifying areas of ACC, was the component regression analysis (Figure 5f, g; Figure 6f, g) carried out over a wider spectral region than the cluster analysis. Although the reference spectra used in the analysis were obtained under different conditions to those of the spatially resolved μ-FTIR maps there is a good level of spatial correlation between the high intensity areas of the ν_2_: ν_4_ maps and the high intensity areas of the ACC component regression maps (Figures 5f, 6f), and also between the low intensity areas of the ν_2_: ν_4_ maps and the high intensity areas of the calcite component regression maps (Figures 5g, 6g). These three independent methods of data analysis have identified broadly similar distributions of areas that appear to correspond to ACC- and calcite-rich regions. There appear to be more similarities between the 100 × 100 μm maps than there are between the whole-granule maps. This no doubt reflects the lower resolution of the latter. Using the intensity maps as guides, we selected several points that were dominated by either ACC or calcite and extracted their spectra (Figure 7). The points identified as ACC-rich have relatively high ν_2_ peaks compared to the ν_4_ peaks, consistent with our method of identifying ACC and other reported ACC FTIR spectra (e.g. [15,38]). Unlike these previous spectra, the ν_2_ band for the ACC-rich regions is also higher in intensity compared to the ν_3_ band and there is no peak corresponding to the ν_1_ band at c. 1084 cm^−1^.

We had hoped to use the μ-FTIR data to map out the distribution of bands related to organic compounds. After demineralising samples of milky fluid obtained from the calciferous gland, Gago-Duport et al. [38] observed peaks at c. 1654 cm^−1^ (amide I), 1540 cm^−1^ (amide II) and c. 1100 cm^−1^ (polysaccharides). Because we detected amino acids in the milky fluid (Table 1) and granules (Tables 4 and 5) and also observed peaks in the milky fluid bulk FTIR spectra that correspond to organic molecules (Figure 1), but not in the bulk granule spectra we had hoped that we might observe such peaks in spatially resolved spectra obtained from the ACC-rich regions of the granules by μ-FTIR mapping. However, these spectra also showed no conclusive evidence for the presence of amide I and polysaccharide species in our mapped samples. There was a wide, variably shaped peak in the range 1570–1350 cm^−1^ in all our spectra taken on the granules. This wavenumber range includes the ν_3_ calcite band at 1420–1470 cm^−1^ and also the amide II (1540 cm^−1^) and lipid/amide III band (1450 cm^−1^) [62]. Point analyses on the resin used to produce the thin sections indicate that contamination from the resin is unlikely to be contributing to this peak. However, to date we have been unable to deconvolute this mixed component peak. It seems that the amino acids detected in our bulk analysis are present in concentrations that are too low to allow their distribution to be mapped out by the μ-FTIR mapping used here.

The elemental mapping of the fresh granules revealed concentric zoning of the style observed in our previous studies [40,41] (e.g. Figures 5h, 6h, 6i). There appeared to be no spatial correlation between the elemental distributions, imaged by the EPMA, and the distribution of ACC within the granules, as determined by the μ-FTIR mapping.

## Conclusions

This study has demonstrated, as have previous studies, that earthworm-secreted calcium carbonate contains ACC. Additionally, in this work we have shown that this ACC is highly heterogeneously distributed but also remarkably stable and can persist for several years. We have also shown that granules contain significant concentrations of amino acids and that granule elemental and amino acid concentration can vary significantly between granules. Considering bulk composition we were unable to identify a dominant controlling factor to account for the ACC in the granules, though significant correlations suggest a link between mass of ACC and amino acid concentration. Using μ-FTIR mapping we have demonstrated the spatial heterogeneity of ACC within granules and, by coupling this to compositional mapping of the same granules, shown that there is no clear correlation between ACC and elemental distribution. We were unable to determine the distribution of organic molecules in the granules using μ-FTIR mapping, possibly due to their low concentration against a background of calcium carbonate. To take our understanding of this system further requires either the isolation of volumes of granules with differing ACC contents for analysis of their organic molecular composition or spatially explicit mapping of the organic molecules present in the granules, perhaps by Time-of-Flight Secondary Ion Mass spectrometry (TOF-SIMS) together with further investigation into the composition of the milky fluid.

## Additional files

Additional file 1: Table S1. Listing the properties of the soils in which earthworms were cultivated. Additional file 2:
**Excel file that contains the FTIR ACC calibration curve and representative FTIR spectra for calcite and ACC.**
Additional file 3: Table S2. Listing the composition of the bulk granules as determined by XRD. Additional file 4: Figure S1. Figure showing the XRD traces for the bulk granules. Additional file 5:
**PowerPoint file that contains all the μFTIR maps gathered for this study.**
