# Supplementary material for: Biomineralisation by earthworms – an investigation into the stability and distribution of amorphous calcium carbonate
Source: Geochem Trans. 2015 Apr 28;16:4. doi: 10.1186/s12932-015-0019-z (PMC4441739; doi:10.1186/s12932-015-0019-z)
Supplement: Additional file 1: Table S1. — Listing the properties of the soils in which earthworms were cultivated. [file 12932_2015_19_MOESM1_ESM.docx]

**Table S1.** Mean chemical characteristics of the < 250 µm soils used in the granule-producing experiments reported in [44] expressed in terms of oven dry soil (n = 3 + standard error except for elemental composition where n = 1). Granules extracted from these soils were analysed in the current study.

| Name^1^ | Sample  site | pH | LOI^3^ | WHC^4^ | CEC^5^ |  | Elemental composition^6^  / wt % | | |  |  |  |  |  |  | Exchangeable ions^7^ / mg kg^-1^ | |  |  |  |  |  |  |
| --- | --- | --- | --- | --- | --- | --- | --- | --- | --- | --- | --- | --- | --- | --- | --- | --- | --- | --- | --- | --- | --- | --- | --- |
|  |  |  |  |  |  |  | Al_2_O_3_ | CaO | Fe_2_O_3_ | K_2_O | MgO | Na_2_O | P_2_O_5_ | SiO_2_ |  | Al | Ca | Fe | K | Mg | Na | P | Sr |
| Coombe Complex | SU625733 | 7.8 + 0.0 | 10.2 + 0.1 | 54.7 + 1.8 | 17.2 + 0.4 |  | 5.8 | 35.9 | 3.3 | 1.3 | 3.0 | 0.1 | 0.3 | 40.9 |  | 1.1+ 0.1 | 1933.6 + 133.6 | 0 | 88.2 + 5.9 | 59.3 + 2.4 | 12.0 + 2.8 | 36.6 + 0.2 | 4.90 + 0.3 |
| Frilsham | SU596726 | 7.0 + 0.0 | 7.7 + 0.1 | 48.7 + 1.9 | 16.6 + 0.2 |  | 7.2 | 0.7 | 3.5 | 1.7 | 0.4 | 0.3 | 0.4 | 77.9 |  | 1.6 + 0.0 | 3401.2 + 9.2 | 0 | 142.5 + 0.3 | 78.1 + 0.2 | 9.3 + 0.8 | 50.2 + 0.7 | 8.35 + 0.1 |
| Hamble | SU618702 | 7.9 + 0.0 | 4.2 + 0.0 | 40.8 + 0.8 | 10.7 + 0.1 |  | 7.6 | 1.3 | 2.9 | 2.0 | 0.6 | 0.4 | 0.2 | 80.3 |  | 1.3 + 0.0 | 2597.4 + 15.3 | 0 | 166.2 + 1.1 | 35.0 + 0.2 | 5.9 + 1.1 | 28.3 + 0.1 | 7.22 + 0.1 |
| Kettering | N/A^2^ | 7.4 + 0.0 | 8.8 + 0.1 | 53.8 + 1.0 | 24.5 + 0.5 |  | 13.2 | 1.9 | 7.0 | 2.2 | 1.0 | 0.2 | 0.2 | 65.5 |  | 2.1 + 0.1 | 4948.0 + 14.0 | 0.2 + 0.0 | 196.9 + 1.6 | 125.6 + 0.4 | 20.1 + 0.8 | 10.7 + 0.4 | 14.3 + 0.1 |
| Neville | SU765754 | 5.4 + 0.0 | 11.2 + 0.1 | 52.0 + 0.1 | 12.2 + 0.7 |  | 5.7 | 0.6 | 3.9 | 1.7 | 0.4 | 0.2 | 0.8 | 76.1 |  | 1.3 + 0.0 | 2533.7 + 5.4 | 0.2 + 0.0 | 473.2 + 3.4 | 262.4 + 0.4 | 13.3 + 1.6 | 102.9 + 1.0 | 8.43 + 0.0 |
| Parkgate | SU601684 | 5.6 + 0.0 | 8.5 + 0.1 | 61.0 + 2.8 | 15.4 + 0.3 |  | 9.0 | 0.4 | 3.6 | 2.2 | 0.7 | 0.3 | 0.4 | 75.0 |  | 1.3 + 0.0 | 2490.9 + 3.6 | 0.3 + 0.0 | 478.0 + 3.5 | 303.8 + 0.4 | 19.2 + 0.5 | 79.0 + 0.1 | 16.8 + 0.1 |
| Soil Science | SU731718 | 6.5 + 0.0 | 19.2 + 0.2 | 71.2 + 1.0 | 37.4 + 0.1 |  | 8.0 | 1.5 | 5.3 | 1.6 | 0.6 | 0.1 | 0.3 | 66.8 |  | 89.9 + 0.8 | 5515.8 + 17.2 | 88.8 + 1.7 | 455.2 + 11.7 | 312.7 + 1.5 | 307.8 + 1.9 | 20.4 + 0.6 | 21.7 + 0.0 |
| St Albans Field | SU603716 | 5.1 + 0.0 | 10.6 + 0.0 | 54.2 + 1.0 | 13.2 + 0.2 |  | 4.6 | 0.4 | 1.9 | 1.1 | 0.2 | 0.1 | 0.5 | 81.2 |  | 16.2 + 0..1 | 2402.2 + 3.9 | 3.9 + 0.1 | 262.1 + 1.8 | 72.4 + 0.1 | 6.7 + 0.2 | 82.3 + 0.8 | 8.74 + 0.0 |
| St Albans Wood | SU602716 | 4.3 + 0.0 | 90.2 + 1.0 | 143.2 + 2.8 | 35.1 + 0.1 |  | 5.7 | 2.1 | 2.2 | 1.1 | 0.3 | 0.0 | 0.5 | 42.5 |  | 2.2 + 0.0 | 5817.0 + 45.7 | 0.2 + 0.0 | 400.6 + 2.6 | 292.7 + 2.4 | 43.1 + 0.4 | 34.9 + 0.6 | 20.9 + 0.3 |
| Tidmarsh | SU626706 | 7.2 + 0.0 | 5.7 + 0.1 | 39.2 + 0.8 | 14.7 + 0.6 |  | 6.8 | 0.6 | 2.4 | 1.8 | 0.4 | 0.4 | 0.3 | 81.3 |  | 1.7 + 0.2 | 3324.0 + 487.7 | 0.1 + 0.0 | 244.7 + 33.6 | 57.8 + 8.5 | 12.8 + 1.4 | 44.0 + 0.1 | 8.55 + 1.3 |
| Wilderness | SU738715 | 6.1 + 0.0 | 27.6 + 0.1 | 78.1 + 1.4 | 42.4 + 0.1 |  | 7.6 | 2.1 | 3.8 | 1.4 | 0.4 | 0.2 | 0.5 | 62.6 |  | 2.7 + 0.1 | 6556.0 + 7.8 | 0.3 + 0.01 | 307.1 + 0.9 | 319.7 + 0.3 | 44.3 + 0.3 | 15.4 + 0.2 | 42.9 + 0.2 |

^1^These correspond to the soil series from which the sample was taken (Jarvis, 1968) except for “Soil Science” and “Wilderness” which were sampled on the University of Reading campus; ^2^Obtained commercially from Broughton Loam and Turf Management, Kettering, UK; ^3^Loss on ignition, %; ^4^Water holding capacity, g_H2O_ g^-1^_soil_; ^5^Cation exchange capacity, cmol_c_ kg^-1^ (Hendershot and Duquette, 1986); ^6^By X-ray fluorescence, normalised to 100 %; ^7^Method of Hendershot and Duquette (1986) except P which is Olsen P (MAFF, 1986)
