# Supplementary material for: Biomineralisation by earthworms – an investigation into the stability and distribution of amorphous calcium carbonate
Source: Geochem Trans. 2015 Apr 28;16:4. doi: 10.1186/s12932-015-0019-z (PMC4441739; doi:10.1186/s12932-015-0019-z)
Supplement: Additional file 3: Table S2. — Listing the composition of the bulk granules as determined by XRD. [file 12932_2015_19_MOESM3_ESM.docx]

Table S2. Rietveld XRD analysis of earthworm secreted granules. Uncertainties relate to the fit of the combination of identified phases to the XRD trace (Coelho, 2003)

| Soil | Weight % mineral |  |  |
| --- | --- | --- | --- |
|  | calcite | quartz | vaterite |
| Frilsham | 92.36 ± 0.25 | 3.209 ± 0.073 | 4.43 ± 0.25 |
| Hamble | 96.23 ± 0.36 | 0.333 ± 0.074 | 3.44 ± 0.36 |
| Kettering | 98.84 ± 0.15 | 1.16 ± 0.15 | - |
| Neville | 66.3 ± 1.3 | 0.17 ± 0.12 | 33.5 ± 1.3 |
| Parkgate | 98.61 ± 0.33 | 0.416 ± 0.085 | 0.97 ± 0.32 |
| St Albans Field | 99.91 ± 0.12 | 0.09 ± 0.12 | - |
| Soil Science | 99.62 ± 0.29 | 0.27 ± 0.16 | 0.10 ± 0.24 |
| Tidmarsh | 82.8 ± 1.1 | 3.3 ± 1.1 | 13.89 ± 0.60 |

Coelho AA: **Indexing of powder diffraction patterns by iterative use of singular value decomposition.** *J Appl Crystallography* 2003, **36:**86-95.
