# Supplementary material for: Biomineralisation by earthworms – an investigation into the stability and distribution of amorphous calcium carbonate
Source: Geochem Trans. 2015 Apr 28;16:4. doi: 10.1186/s12932-015-0019-z (PMC4441739; doi:10.1186/s12932-015-0019-z)

Fig. S1. XRD traces for bulk granule analysis. F= Frilsham, H = Hamble, K = Kettering, N = Neville, PG = Parkgate, SA = St Albans, SS = Soil Science, T = Tidmarsh. CC indicates a peak associated with calcite, Vat with vaterite.


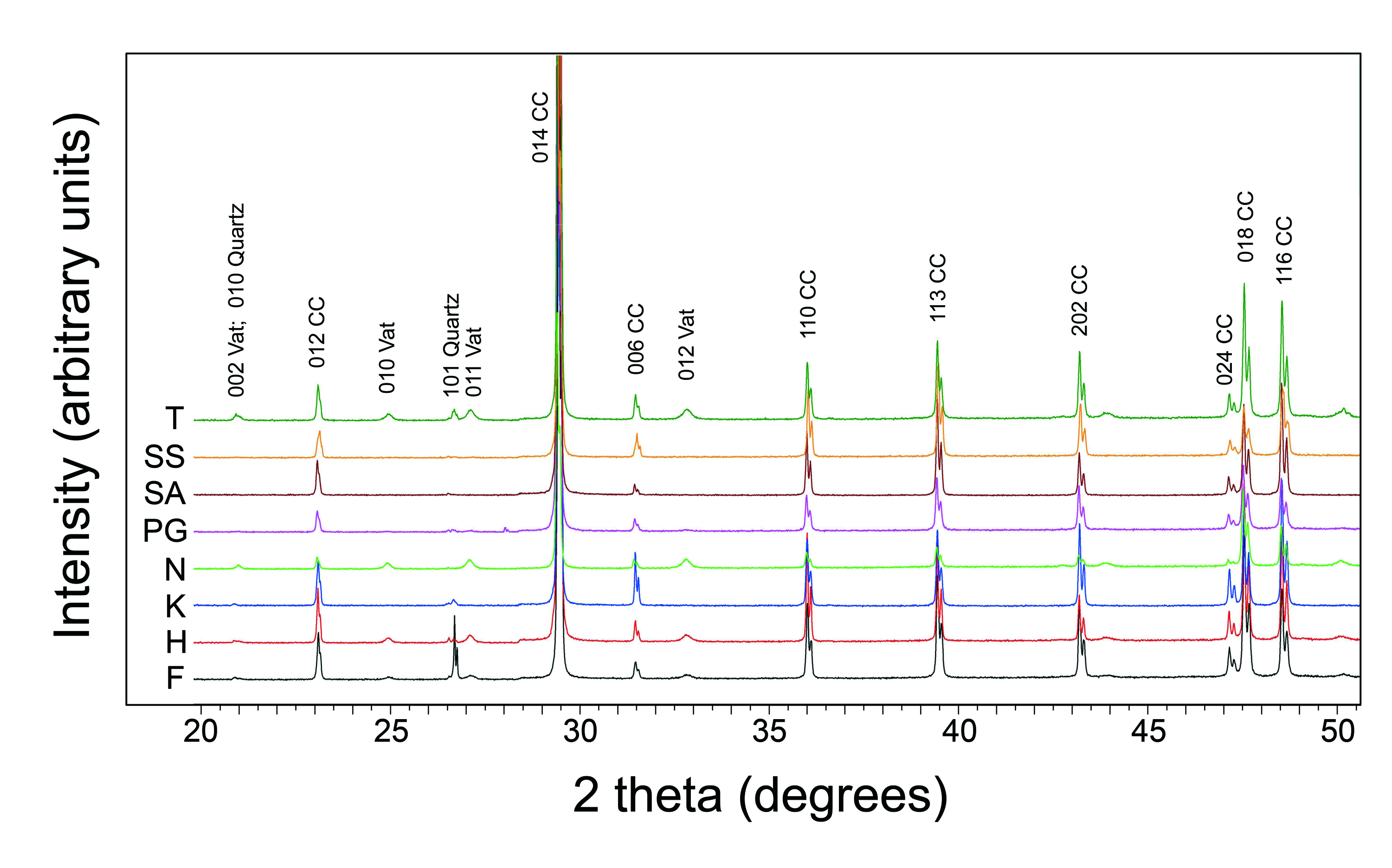

Supplement: Additional file 4: Figure S1. — Figure showing the XRD traces for the bulk granules. [file 12932_2015_19_MOESM4_ESM.docx]
