# Supplementary material for: Biomineralisation by earthworms – an investigation into the stability and distribution of amorphous calcium carbonate
Source: Geochem Trans. 2015 Apr 28;16:4. doi: 10.1186/s12932-015-0019-z (PMC4441739; doi:10.1186/s12932-015-0019-z)
Supplement: Additional file 5: — PowerPoint file that contains all the μFTIR maps gathered for this study. [file 12932_2015_19_MOESM5_ESM.pptx]

## Slide 1
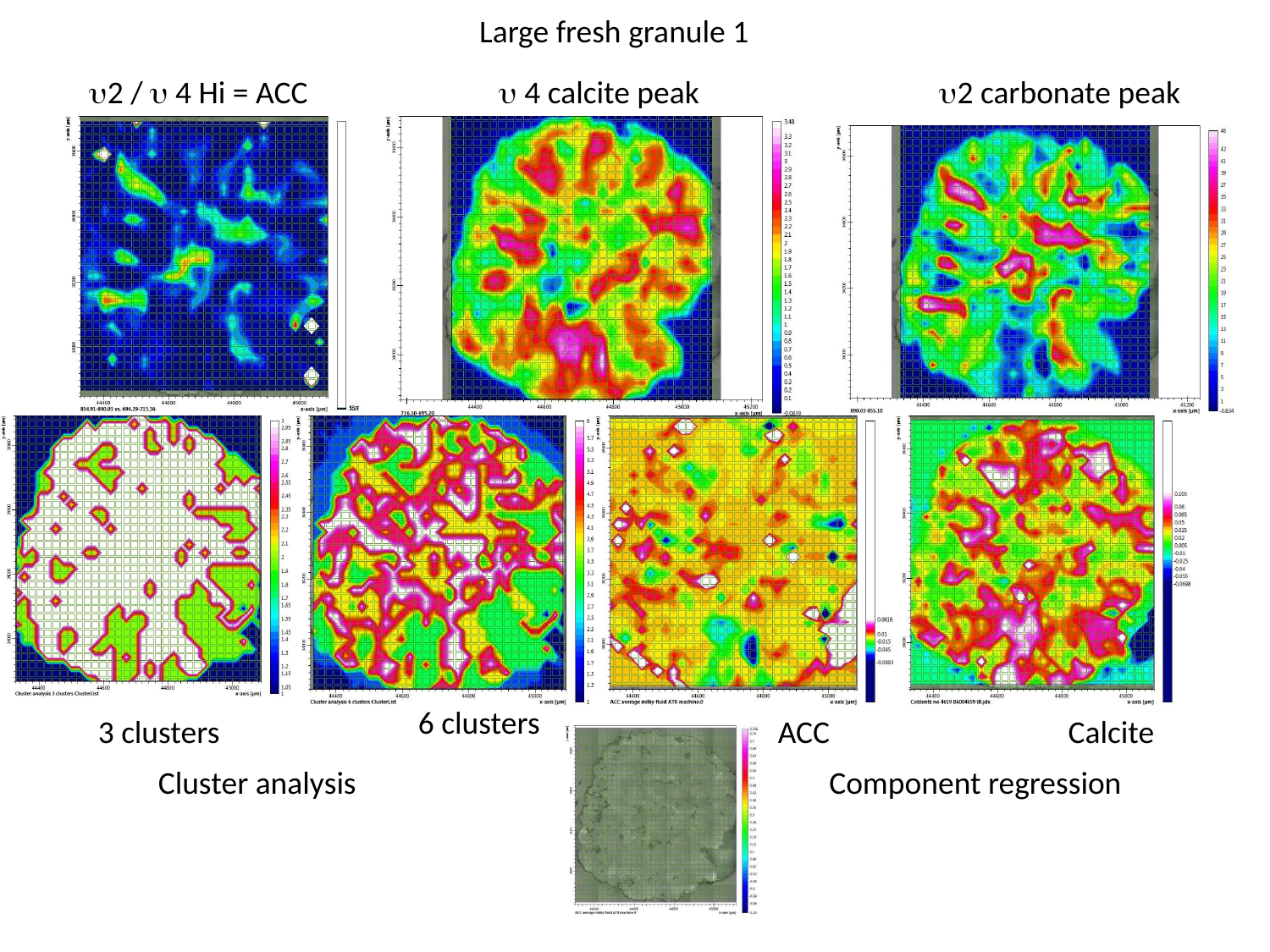

Large fresh granule 1
2 /  4 Hi = ACC
 4 calcite peak
2 carbonate peak
#
6 clusters
3 clusters
ACC
Calcite
Cluster analysis
Component regression

## Slide 2
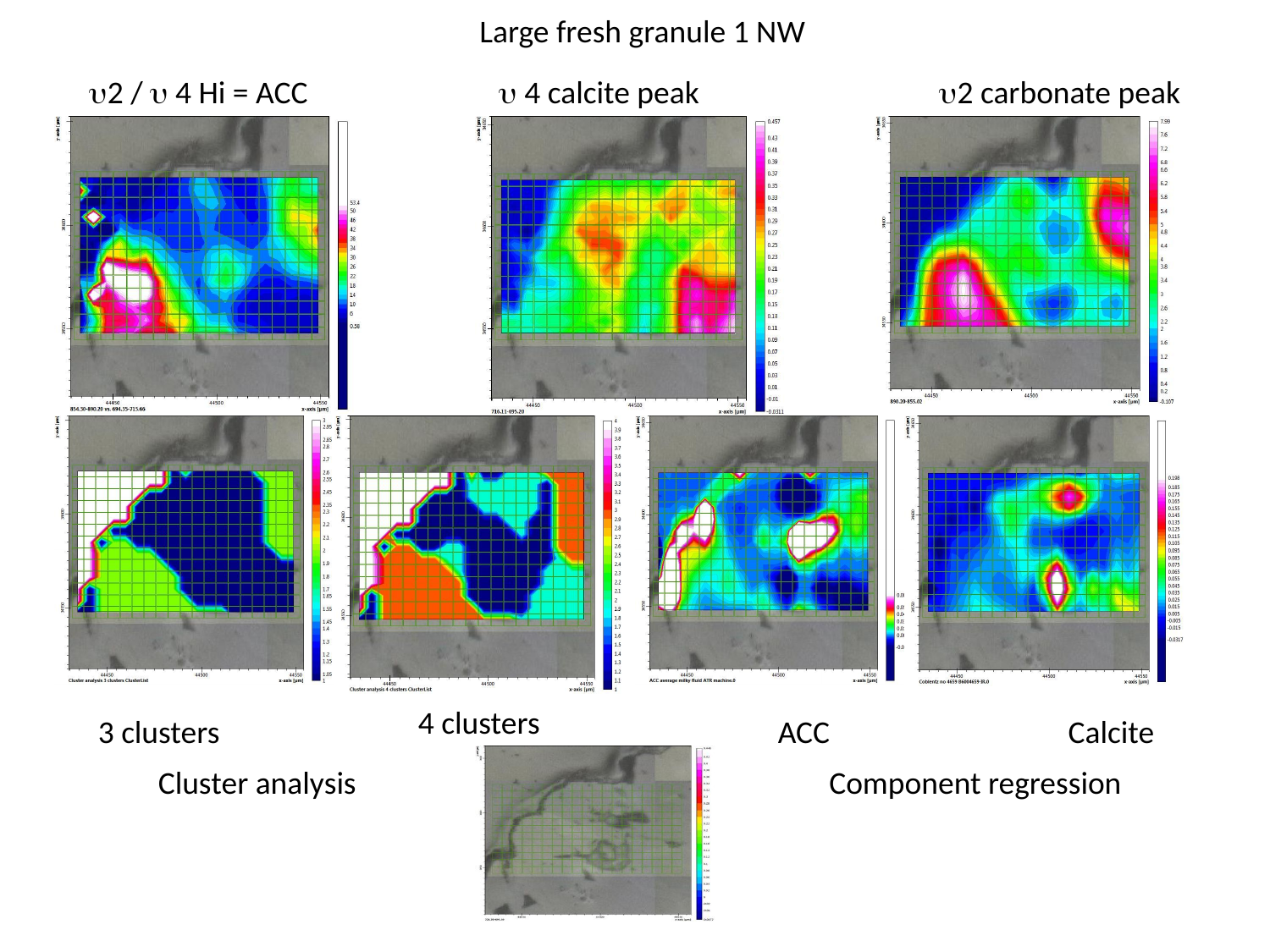

Large fresh granule 1 NW
2 /  4 Hi = ACC
 4 calcite peak
2 carbonate peak
#
4 clusters
3 clusters
ACC
Calcite
Cluster analysis
Component regression

## Slide 3
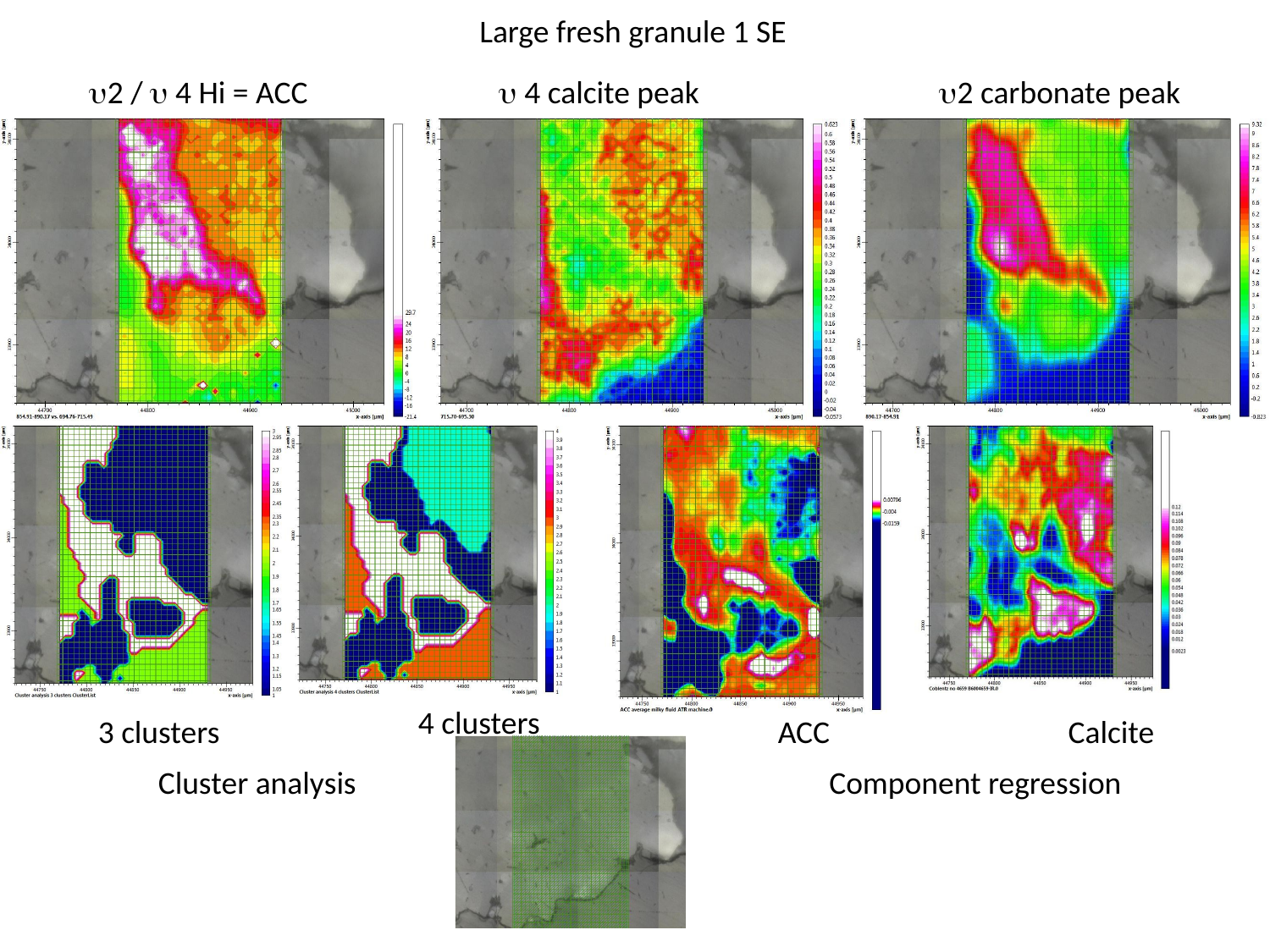

Large fresh granule 1 SE
2 /  4 Hi = ACC
 4 calcite peak
2 carbonate peak
#
4 clusters
3 clusters
ACC
Calcite
Cluster analysis
Component regression

## Slide 4
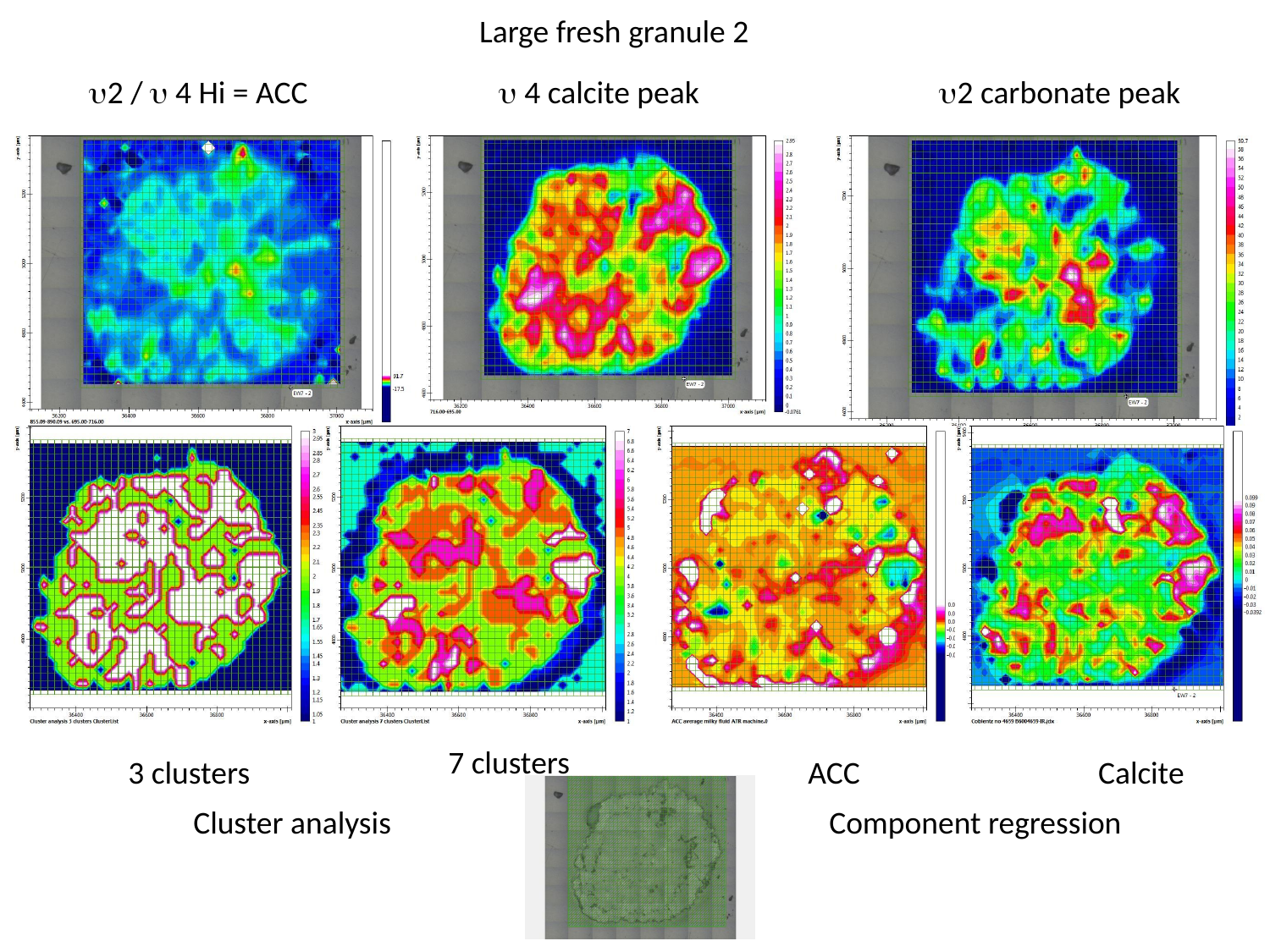

Large fresh granule 2
2 /  4 Hi = ACC
 4 calcite peak
2 carbonate peak
#
7 clusters
3 clusters
ACC
Calcite
Cluster analysis
Component regression

## Slide 5
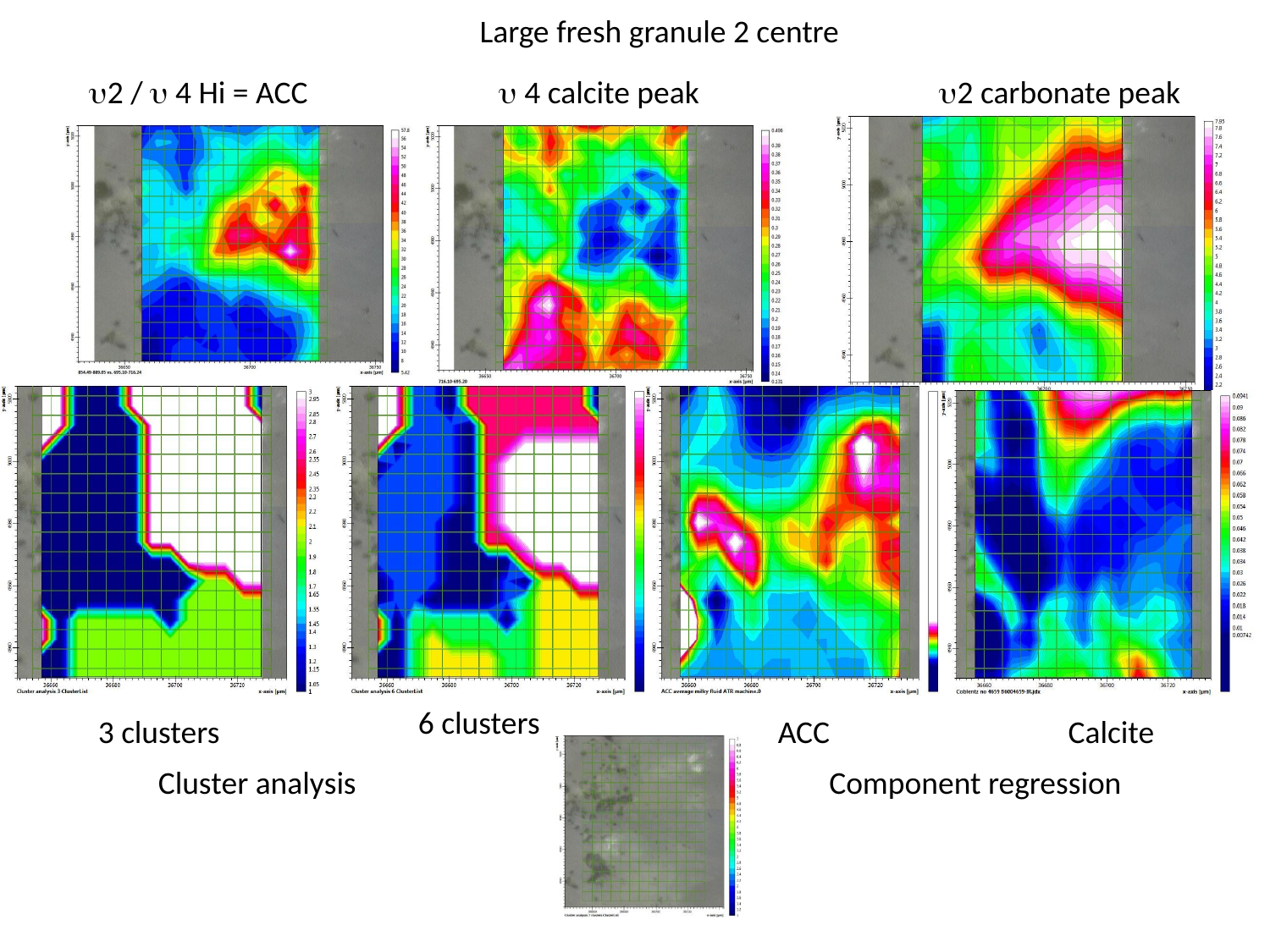

Large fresh granule 2 centre
2 /  4 Hi = ACC
 4 calcite peak
2 carbonate peak
#
6 clusters
3 clusters
ACC
Calcite
Cluster analysis
Component regression

## Slide 6
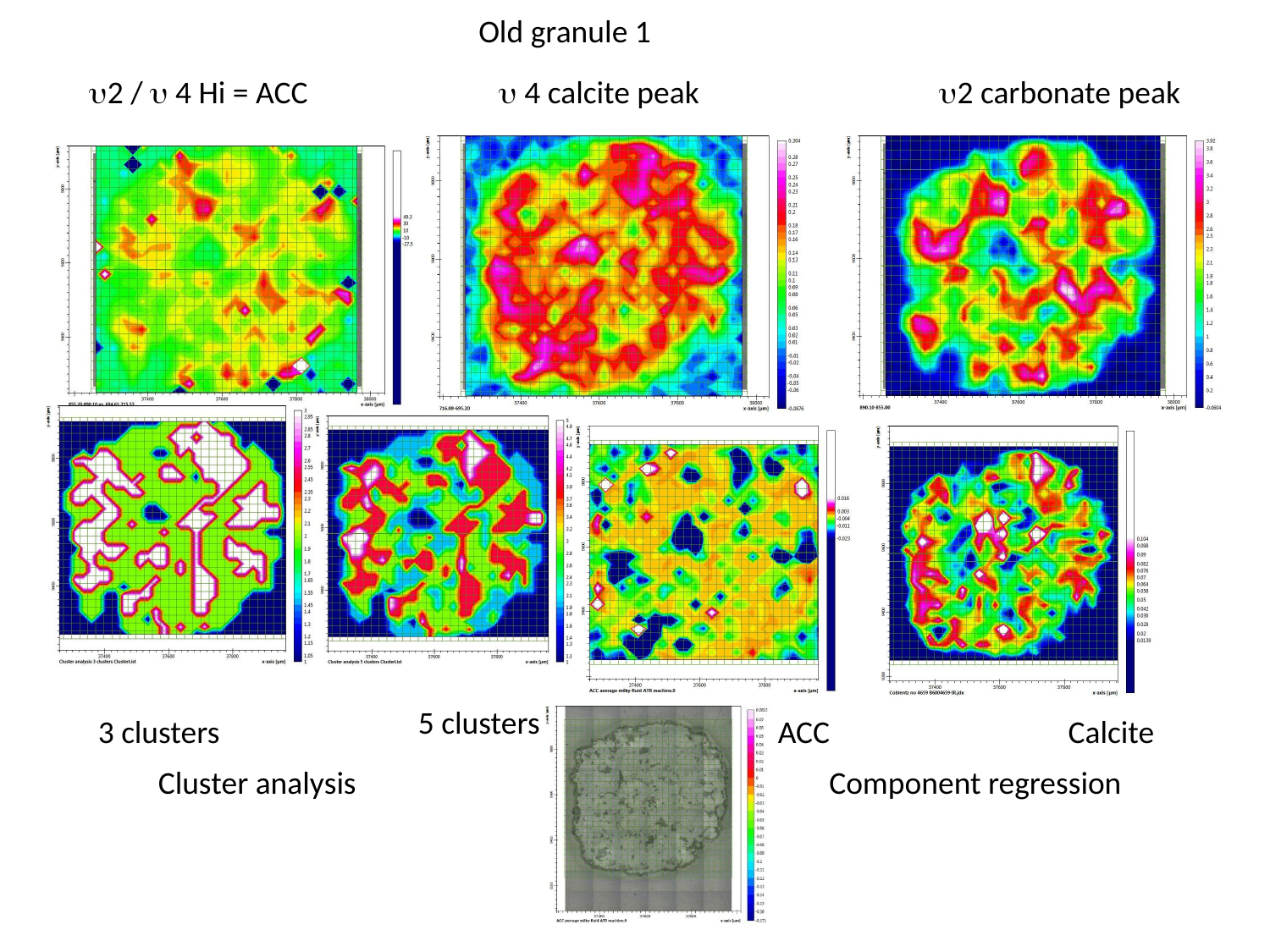

Old granule 1
2 /  4 Hi = ACC
 4 calcite peak
2 carbonate peak
#
5 clusters
3 clusters
ACC
Calcite
Cluster analysis
Component regression

## Slide 7
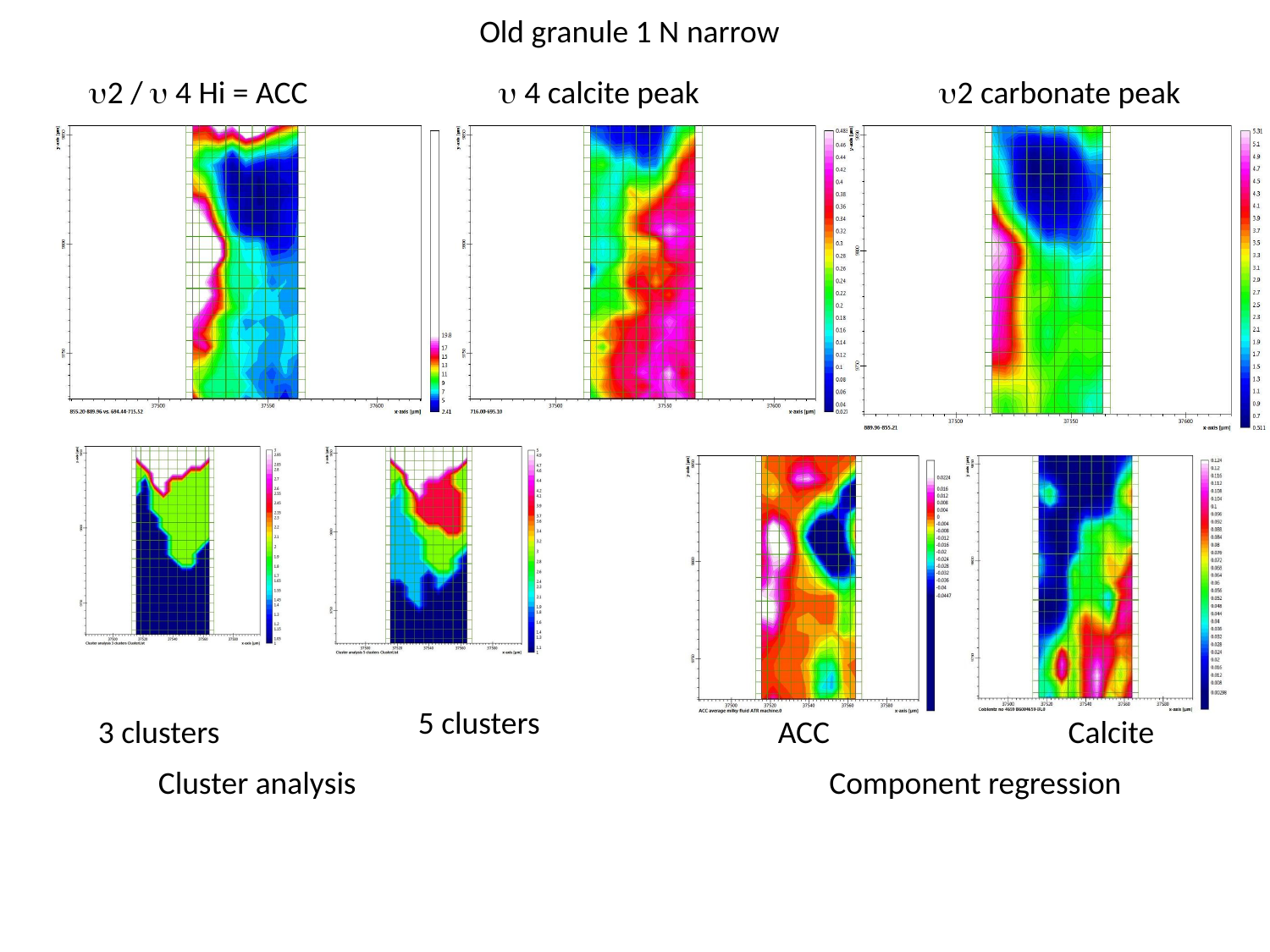

Old granule 1 N narrow
2 /  4 Hi = ACC
 4 calcite peak
2 carbonate peak
#
5 clusters
3 clusters
ACC
Calcite
Cluster analysis
Component regression

## Slide 8
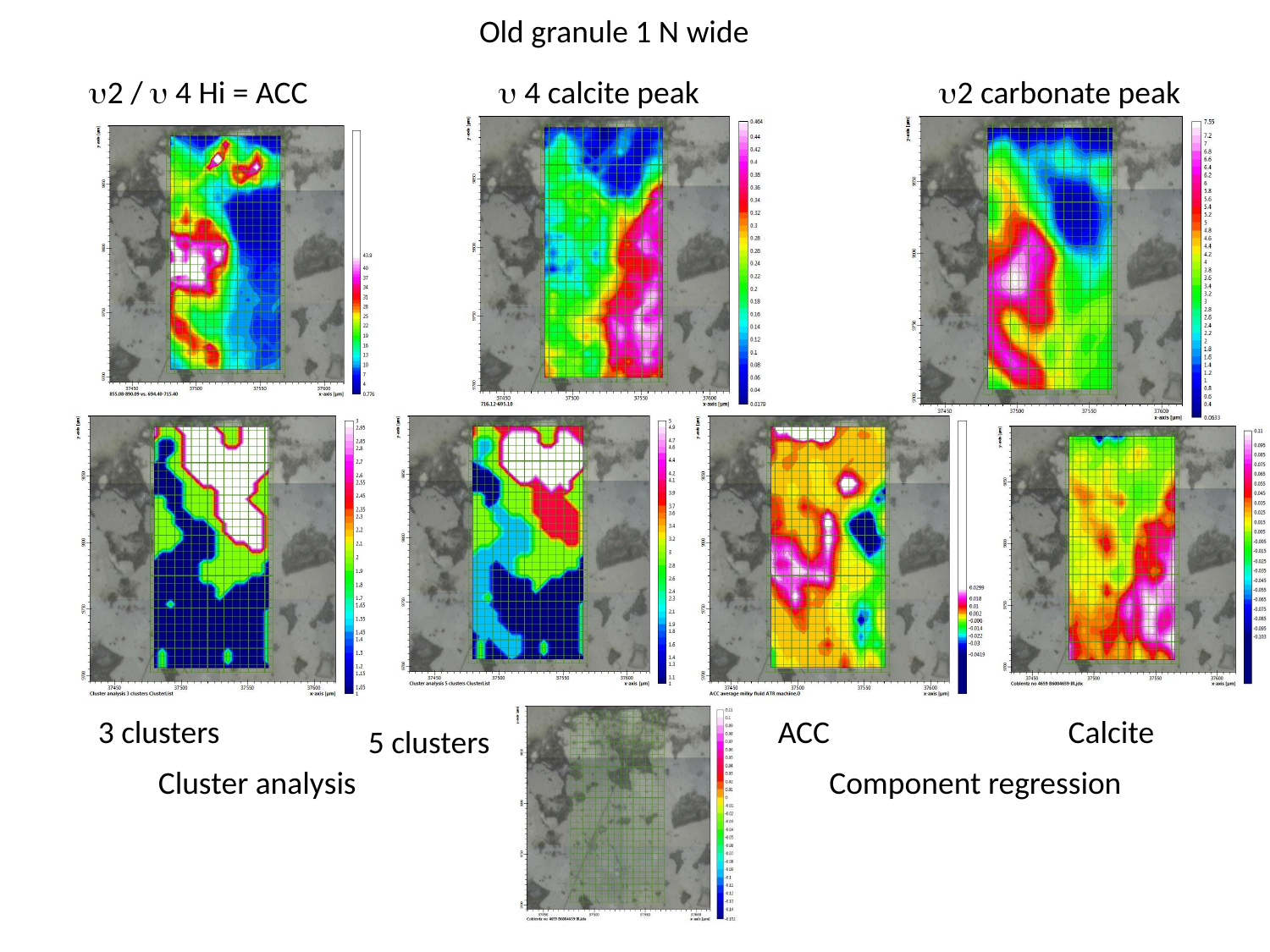

Old granule 1 N wide
2 /  4 Hi = ACC
 4 calcite peak
2 carbonate peak
#
3 clusters
ACC
Calcite
5 clusters
Cluster analysis
Component regression

## Slide 9
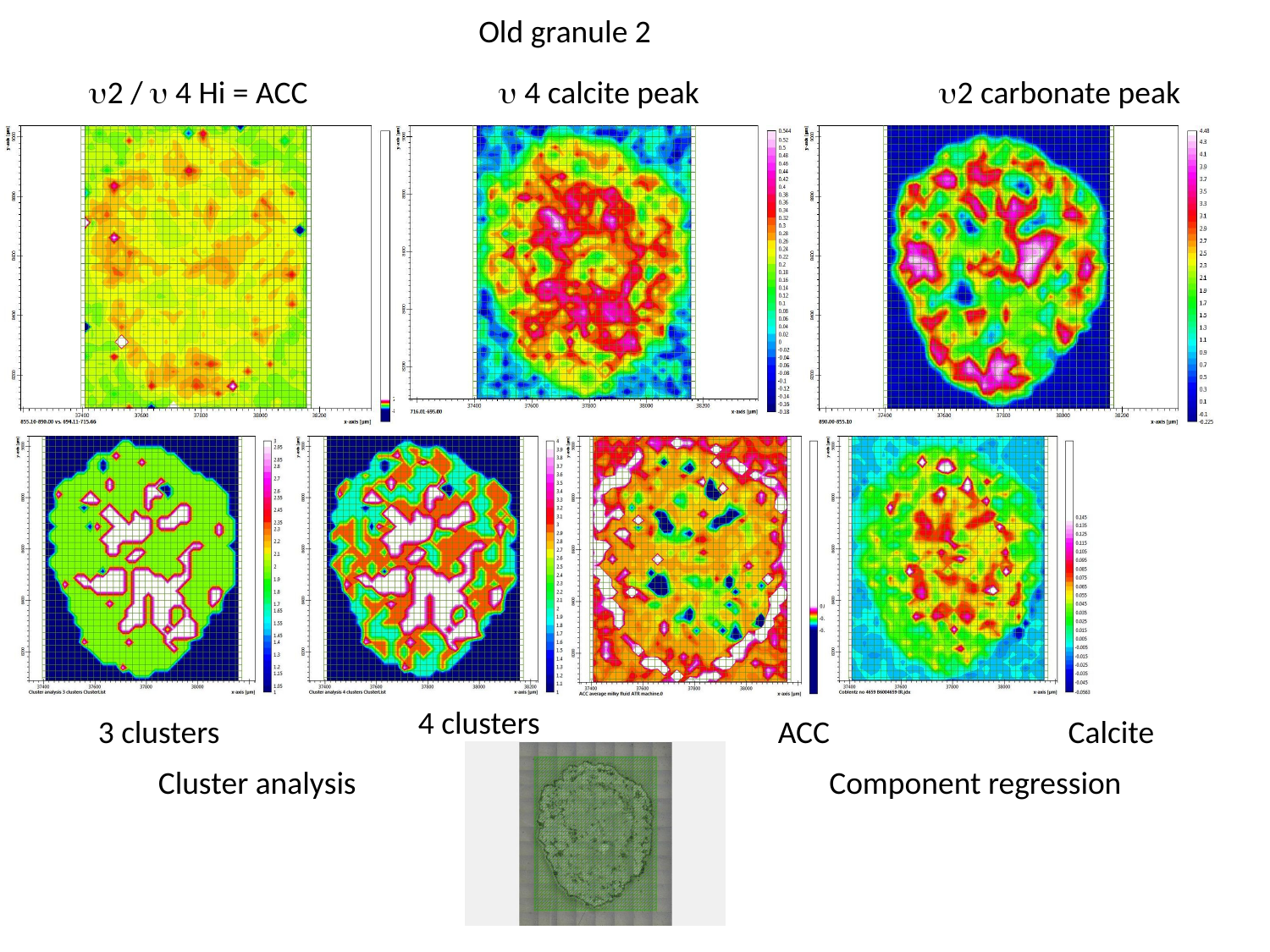

Old granule 2
2 /  4 Hi = ACC
 4 calcite peak
2 carbonate peak
#
4 clusters
3 clusters
ACC
Calcite
Cluster analysis
Component regression

## Slide 10
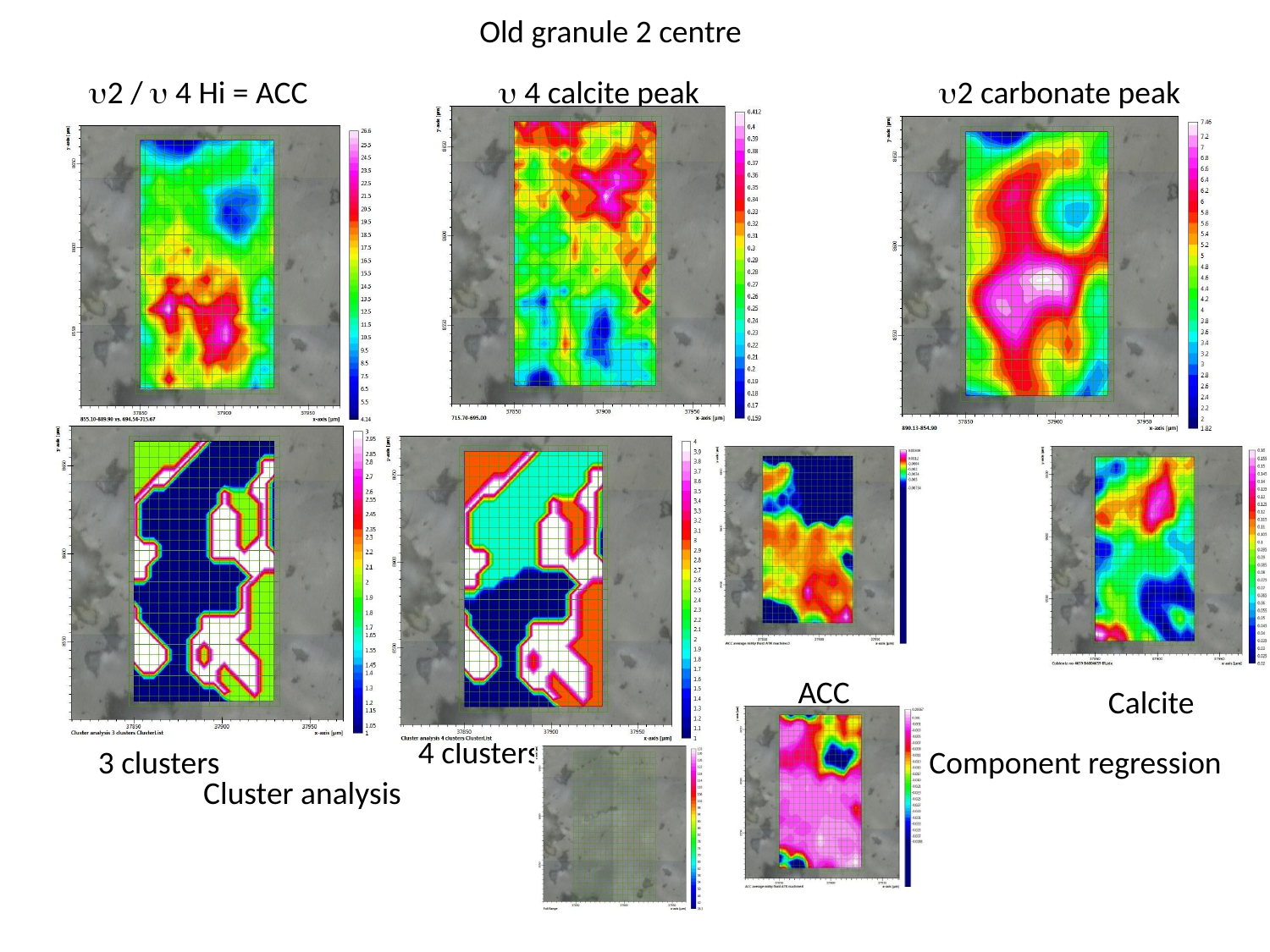

Old granule 2 centre
2 /  4 Hi = ACC
 4 calcite peak
2 carbonate peak
#
ACC
Calcite
4 clusters
3 clusters
Component regression
Cluster analysis
